# Switchable Anion Exchange in Polymer-Encapsulated APbX_3_ Nanocrystals Delivers Stable All-Perovskite White Emitters

**DOI:** 10.1021/acsenergylett.1c01232

**Published:** 2021-07-22

**Authors:** Muhammad Imran, Binh T. Mai, Luca Goldoni, Matilde Cirignano, Houman Bahmani Jalali, Francesco Di Stasio, Teresa Pellegrino, Liberato Manna

**Affiliations:** † Nanochemistry, Istituto Italiano di Tecnologia, Via Morego 30, 16163 Genova, Italy; ‡ Nanomaterials for Biomedical Applications, Istituto Italiano di Tecnologia, Via Morego 30, 16163 Genova, Italy; § Analytical Chemistry Lab, Istituto Italiano di Tecnologia, Via Morego 30, 16163 Genova, Italy; ∥ Photonic Nanomaterials, Istituto Italiano di Tecnologia, Via Morego 30, 16163 Genova, Italy; ⊥ Dipartimento di Chimica e Chimica Industriale, Universitàdegli Studi di Genova, Via Dodecaneso 31, 16146 Genova, Italy

## Abstract

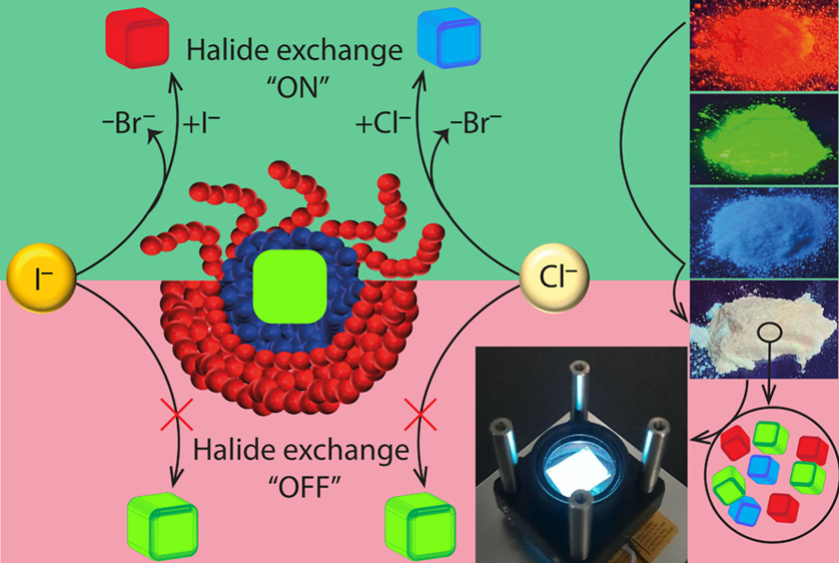

We report a one-step synthesis of halide perovskite nanocrystals embedded in amphiphilic polymer (poly(acrylic acid)-*block*-poly(styrene), PAA-*b*-PS) micelles, based on injecting a dimethylformamide solution of PAA-*b*-PS, PbBr_2_, ABr (A = Cs, formamidinium, or both) and “additive” molecules in toluene. These bifunctional or trifunctional short chain organic molecules improve the nanocrystal–polymer compatibility, increasing the nanocrystal stability against polar solvents and high flux irradiation (the nanocrystals retain almost 80% of their photoluminescence after 1 h of 3.2 w/cm^2^ irradiation). If the nanocrystals are suspended in toluene, the coil state of the polymer allows the nanocrystals to undergo halide exchange, enabling emission color tunability. If the nanocrystals are suspended in methanol, or dried as powders, the polymer is in the globule state, and they are inert to halide exchange. By mixing three primary colors we could prepare stable, multicolor emissive samples (for example, white emitting powders) and a UV-to-white color converting layer for light-emitting diodes entirely made of perovskite nanocrystals.

Metal halide perovskite (MHP) nanocrystals (NCs) are promising materials for light emitting technologies.^1−4^ NCs with various compositions (AMX_3_, A = Cs, CH_3_NH_3_, or HC(NH)NH_2_, M = Pb, Sn and X = Cl, Br, I) can be prepared easily,^5−10^ and their emission color is tunable across the ultraviolet–visible spectrum and beyond, by alloying, doping, or anion/cation exchange.^6,8,11−14^ These NCs are coated with surfactant molecules that stabilize them in nonpolar or moderately polar organic solvents.^15−18^ Yet, the NCs have a poor stability against moisture, polar solvents, and long-time exposure to irradiation. Also, when MHP NCs of different emission colors are deposited together in solid films or are mixed in a colloidal suspension, they undergo halide ion exchange. This inter-NCs reactivity limits the use of MHP NCs as multicolor emitters, for example, in white light emission.^3,19−21^ So far, white light emission was achieved mainly by blending green emitting perovskite NCs with organic dyes or metal chalcogenides NCs. Similarly, in down-converting white light emitting devices white emission was achieved by combining green emitting perovskite NCs with a commercial UV-LED chip and a nonperovskite based red phosphor.^22−31^

Strategies have been designed to prevent anion exchange and/or to stabilize MHP NCs against polar solvents, including encapsulation of NCs in matrixes made of metal oxides (e.g., PbSO_4_, SiO_2_, TiO_2_, Al_2_O_3_), inorganic salts (SrBr_2_), a mixture of metal oxides and inorganic salts, metal organic hybrids (e.g., metal organic frameworks), and polymers.^23,24,32−46^ Polymers are appealing as they can switch their chains from an extended coil state to a collapsed globule state when the characteristics of the solvent change. In its globule state, a polymer can act as a protecting layer that shields the NCs from their surroundings, making them unreactive. In its coil state, a polymer stretches in solution and partially exposes the NC’s surface to the surrounding media, thus allowing a series of chemical reactions with the NCs, hence making them “reactive”. The switch from the globule to the coil phase of a polymer is solvent-dependent and can be properly tuned. This solvent-driven unique feature of certain classes of polymers to reversibly switch the behavior of MHP NCs from unreactive to reactive, especially toward anion exchange reactions, has remained unexplored until now. Polymeric micelles have also been used as templates to synthesize inorganic NCs with low polydispersity and a stable surface coating.^47−50^ Polymers with hydrophobic characteristics are ideal choices to protect MHP NCs from polar solvents and have been widely tested in this regard.^51−57^ For example, previous studies have shown that embedding CsPbBr_3_ NCs in different polymeric matrices significantly improves the stability of such NCs.^58^ Similarly, various types of polymeric micelles have been exploited as a template to encapsulate, *in situ*, individual MHP NCs.^59−62^ A more detailed discussion of these studies, along with a critical discussion and comparison with the present work, is reported in the SI (see also Table S1). From these studies it emerges that the resistance of the NCs against high volume ratio of polar solvents (and especially short-chain alcohols such as methanol) and high flux irradiation is still limited. Also, most of these preparative approaches are not widely accessible and/or scalable, as they often involve various polymerization steps. Overall, the stability of these NCs under operating conditions that are typical for many applications (high humidity, polar solvents, high flux irradiation, etc.) has yet to be proven satisfactory.

We report here an efficient method to encapsulate APbX_3_ NCs by combining a commercially available diblock copolymer, namely poly(acrylic acid)-*block*-poly(styrene) (PAA-*b*-PS), with short chain “additive molecules” that contain two or more functional groups (for example, carboxylic, phosphonic, amino, or combinations of these). The additive molecules improve the compatibility between the NCs and the block copolymer during the encapsulation process, as they increase the stability of the encapsulated NCs against polar solvents and high flux irradiation. Extensive nuclear magnetic resonance (NMR) analysis on the polymeric micelles encapsulating the NCs proved their core–shell structure, with PAA forming the core of the micelles and PS the outer shell. When the polymer-encapsulated NCs are suspended in nonpolar solvents (for example, toluene), the polymer shell remains in the coil state, and the NCs can undergo halide exchange reactions, allowing color tunability. Instead, when the NCs are dispersed in polar solvents (such as methanol), or dried and stored as powders, the polymer shell is in the globule state, and the surface of the NCs cannot be accessed by chemicals from their surroundings, thereby making them inert toward halide exchange reactions. Based on this property, we could prepare stable multicolor emissive samples retaining their spectral features even after months of storage under air. Such control over the structural and color stability enabled us to prepare white emitting powders fully based on perovskite NCs. The PAA-*b*-PS-encapsulated NCs were very robust against high flux irradiation (3.2 w/cm^2^), retaining over 78% of their photoluminescence (PL) efficiency after 1 h of continuous irradiation under such conditions (this can be considered an accelerated stability test). Finally, the white emitting powder was embedded in a polymeric matrix and a UV-to-white color converting layer for light-emitting diodes entirely made of perovskite NCs was fabricated.

The PAA-*b*-PS-encapsulated NCs were prepared under ambient conditions. PAA-*b*-PS with a molar mass of 5000 and 28000 g·mol^–1^, respectively, was chosen to ensure that the resulting assembled structures are micelles with a PAA core and a PS shell in a nonpolar solvent such as toluene.^63^ As additives, we used short organic molecules carrying two or more functional groups: these were either all acidic in nature or a combination of acidic groups and basic (for example, amino) ones (Figure 1; see discussion later). PAA-*b*-PS-encapsulated APbBr_3_ (A = Cs, formamidinium (FA) or their mixture) NCs were prepared by a ligand assisted reprecipitation route. In a typical synthesis, ABr (CsBr, FABr or their mixture), PbBr_2_ PAA-*b*-PS, and 5-aminopentanoic acid (APAc) as an additive molecule (one of the best working molecules) were separately dissolved in dimethylformamide (DMF) to form stock solutions. PbBr_2_ and FABr were dissolved quickly in DMF, while CsBr had a negligible solubility in it (even under sonication at 60 °C). The addition of PAA-*b*-PS to DMF (at a ratio of 1:20 for Cs to PAA) helped to dissolve CsBr completely. Then, equal molar volumes of ABr and PbBr_2_ stock solutions were mixed with PAA-*b*-PS and APAc stock solutions. This solution was injected dropwise in toluene (a selective solvent for the PS block), triggering at once the formation of polymer micelles and the nucleation and growth of APbBr_3_ NCs in the micelles. After 20 s, the reaction was quenched by adding excess hexane and the NCs were collected by centrifugation. The supernatant was discarded and the precipitate was redispersed in toluene followed by another round of centrifugation and redispersion in toluene.

**Figure 1:**
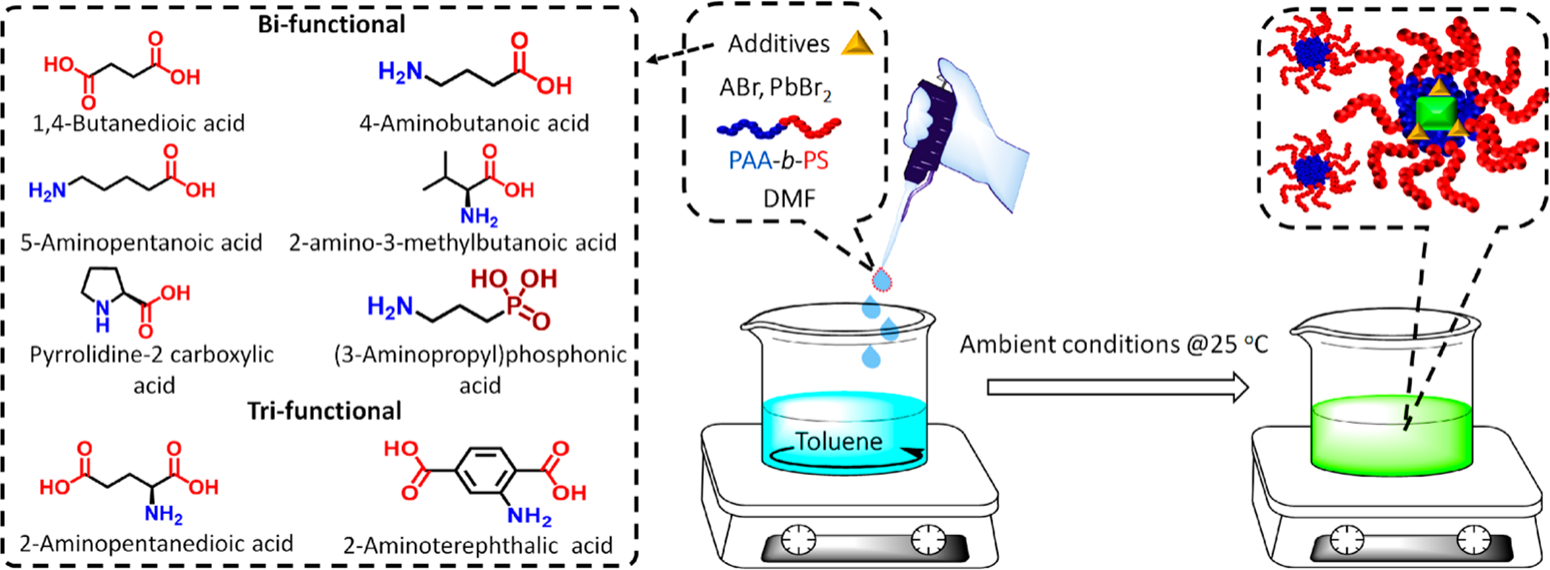
Sketch of the synthesis of APbX_3_ NCs encapsulated in PAA-*b*-PS micelles (right), along with a set of additive molecules that turned out to be successful to increase the stability of such NCs (left). The screening and role of these molecules are discussed in detail later in this work.

The NCs under the transmission electron microscope (TEM) had nearly cubic shapes in all three cases (Figure S1). We carried out dynamic light scattering (DLS) measurements on the PAA-*b*-PS-encapsulated CsPbBr_3_ NCs and on PAA-*b*-PS empty micelles, in toluene. Empty micelles were prepared in the same way as the polymer-encapsulated NCs, except for the addition of metal halide salts (ABr and PbBr_2_) and additive molecules in the DMF solution. The empty micelles and the NCs had hydrodynamic diameters (*d*_H_) of 48 and 80 nm (weighted by intensity), respectively, with a single peak present in the 1–1000 nm range (Figures S2 and S3). The data for the NC sample exclude the presence of large aggregates of NCs, in agreement with TEM analyses. The *d*_H_ values weighted by volume and number were also as small as 51 and 61 nm, confirming the absence of aggregates or large particles (Figures S2 and S3). Similar numbers were found by DLS measurements on PAA-*b*-PS-encapsulated Cs_0.5_FA_0.5_PbBr_3_ and FAPbBr_3_ NCs (Figures S4 and S5). The polydispersity index (PDI) of the empty micelles and PAA-*b*-PS-encapsulated NCs was in the range of 0.06–0.13, confirming their narrow size distribution (Table S2). Based on X-ray diffraction (XRD, Figure S6a), perovskite was the only phase in all samples. Partial or complete substitution of the A-site cation Cs with the larger FA cations was attested by the shift of the XRD peaks toward lower angles (larger cell) compared to the reference CsPbBr_3_ pattern (Figure S6b). In toluene, the NCs had emissions centered at 517 nm (CsPbBr_3_), 527 (Cs_0.5_FA_0.5_PbBr_3_), and 535 nm (FAPbBr_3_) with 18–22 nm line widths (Figures S6c and S7). PL quantum yield (PLQY) values of the samples (in toluene dispersions) were in the 30–61% range and average PL lifetimes were in the 1.6–36 ns range (Figure S8 and Table S3).

On the PAA-*b*-PS-encapsulated APbBr_3_ NCs we have the unique chance to reversibly turn on/off anion exchange: when the NCs were dispersed in toluene, in which the polymer is in the coiled state, anion exchange was allowed and the emission color of the NCs was tuned by dosing the halide reagent, didodecyldimethylammonium chloride (DDACl) or oleylammonium iodide (OLAM-I) for the exchange of bromide with chloride or iodide, respectively (Figure 2a,b; see the Experimental Section in the SI for details). When the NCs were dispersed in methanol, in which the polymer is in a globule state (due to the low solubility of the polymer in this solvent), anion exchange was prevented. This is demonstrated on green emitting PAA-*b*-PS-encapsulated CsPbBr_3_ NCs; see Figure 2b,c and Videos S1 and S2. Even when both chloride and iodide reagents were introduced together in large excess in the methanol dispersion of PAA-*b*-PS-encapsulated CsPbBr_3_ NCs, the initial PL spectral position and PL line width were retained, thus confirming a complete inhibition of anion exchange (Figure 2c). On the other hand, anion exchange could be easily triggered by adding an excess amount of toluene to open the PS-protecting layer. This is demonstrated by adding the chloride reagent in large excess to the methanol dispersion of PAA-*b*-PS-encapsulated CsPbBr_3_ NCs: halide exchange did not occur until the addition of toluene (Video S3). We also tested anion exchange reactions on green emitting PAA-*b*-PS-encapsulated NCs using other reagents, such as ZnI_2_, which is highly soluble in methanol (as opposed to OLAM-I which is weakly soluble) and has been previously used for halide exchange reactions in toluene dispersions of perovskite NCs.^64^ Also in this case, anion exchange was prevented in methanol dispersions (Figure S9-a and Video S4), while it was allowed in toluene (Figure S9-b and Video S4). This proves that the switchable halide exchange behavior of our polymer coated NCs does not depend on the type of halide reagent used and is only related to the coil/globule state of the polymer. The sketches in Figure 2d summarize the halide exchange reactions carried out under the various conditions.

**Figure 2:**
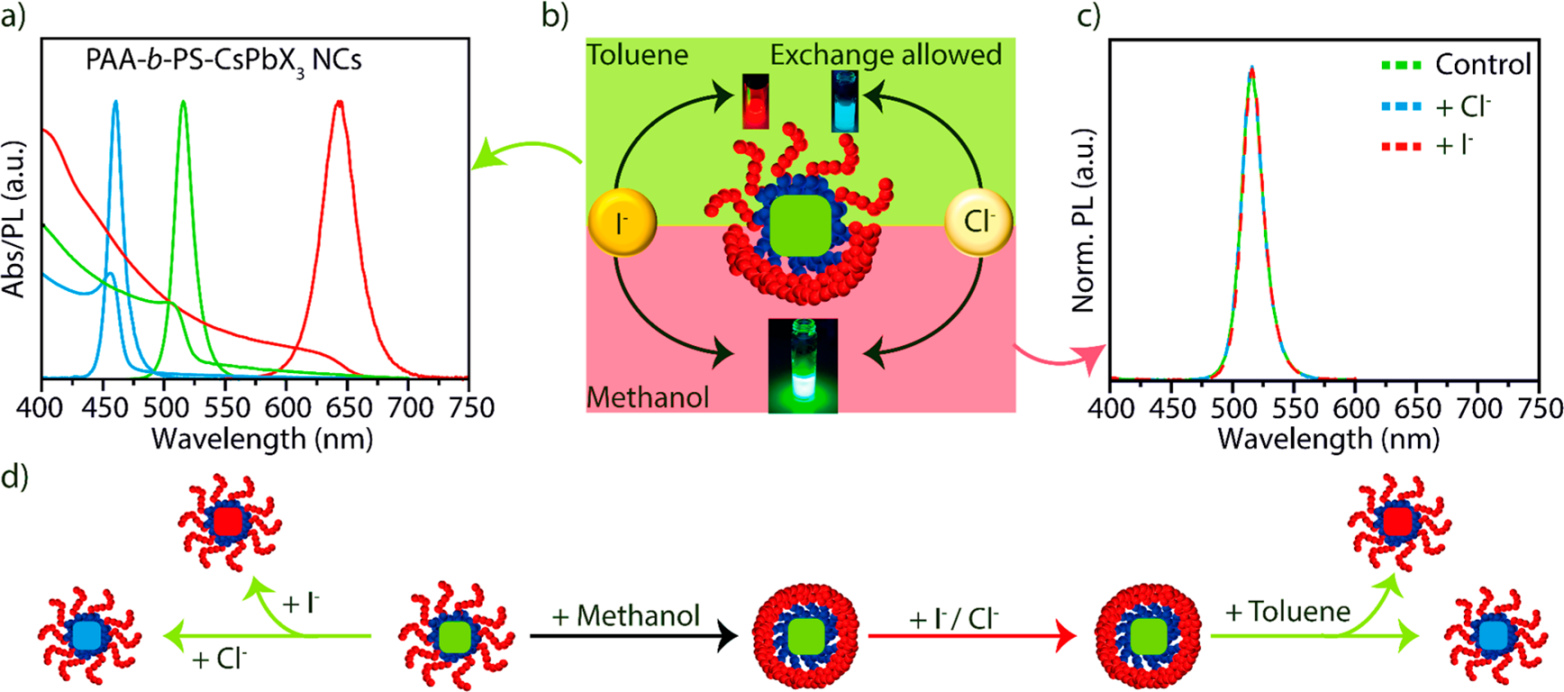
“On demand” color tunability in PAA-*b*-PS-encapsulated CsPbBr_3_ NCs. (a) Optical absorption and PL spectra of the initial CsPbBr_3_ NCs sample dispersed in toluene (green curves) and of the corresponding samples upon the addition of either didodecyldimethylammonium chloride (blue curves) or oleylammonium iodide solutions (red curves). The sketch in (b) illustrates the switchable anion exchange mechanism: in toluene dispersions, the polymer shell is open (coil state) and the color tunability is allowed upon halide addition. In methanol, the polymer shell is closed (globule state); hence anion exchange is inhibited and the pristine color is retained. The latter case is illustrated in panel (c) reporting the PL spectra of the initial CsPbBr_3_ NCs sample and that of the samples after addition of the chloride and iodide salts. (d) Sketches summarizing the halide exchange reactions under the various conditions.

We then assessed the stability of the PAA-*b*-PS-encapsulated NCs in various polar solvents, choosing PAA-*b*-PS-encapsulated CsPbBr_3_ NCs as a representative system. The toluene dispersion of these NCs was added dropwise to methanol at a volume ratio of 1 to 10 (toluene to methanol) under ambient conditions, to induce the globule state of the PS shell, as discussed earlier. After about 30 s, an excess amount of hexane was added and the NCs dispersion was centrifuged. Then, the supernatant was discarded, the precipitate was redispersed in the desired polar solvent (methanol, ethanol, water), and the NCs dispersions were stored under ambient conditions to examine their PL stability over time. The PL spectra recorded over time for PAA-*b*-PS-encapsulated CsPbBr_3_ NCs dispersed in various solvents are reported in Figure S10. The NCs dispersed in toluene and water nearly retained their PL intensity and PL spectral position (Figure S10a,b). In ethanol and methanol, a drop of 18% and 53% in PL intensity was seen after 23 days of aging, while the PL spectral positions remained nearly unchanged (Figure S10c,d). Quantitative data on the drop of PL intensity of the polymer-encapsulated NCs in different solvents are reported in Figure 3a. These are compared with “reference” CsPbBr_3_ NCs that were synthesized using oleic acid and oleylamine as surfactants (following the method of Protesescu et al.^6^), hence not polymer-encapsulated. The PL from this reference sample was immediately quenched as soon as methanol was added to it. A more extended comparison is reported in Figure 3b: we benchmarked the stability in methanol of the PAA-*b*-PS-encapsulated CsPbBr_3_ NCs with various NCs which were prepared following published recipes.^6,65,66^ Again, these NCs were not polymer-encapsulated but they were coated with various ligands, such as Cs-oleate, mixed ligands (Cs-oleate/oleylammonium bromide), and didodecyldimethylammonium bromide (DDABr capped) and were initially dispersed in toluene. As soon as methanol was added, all the NCs samples lost their PL, except for the polymer-encapsulated one.

**Figure 3:**
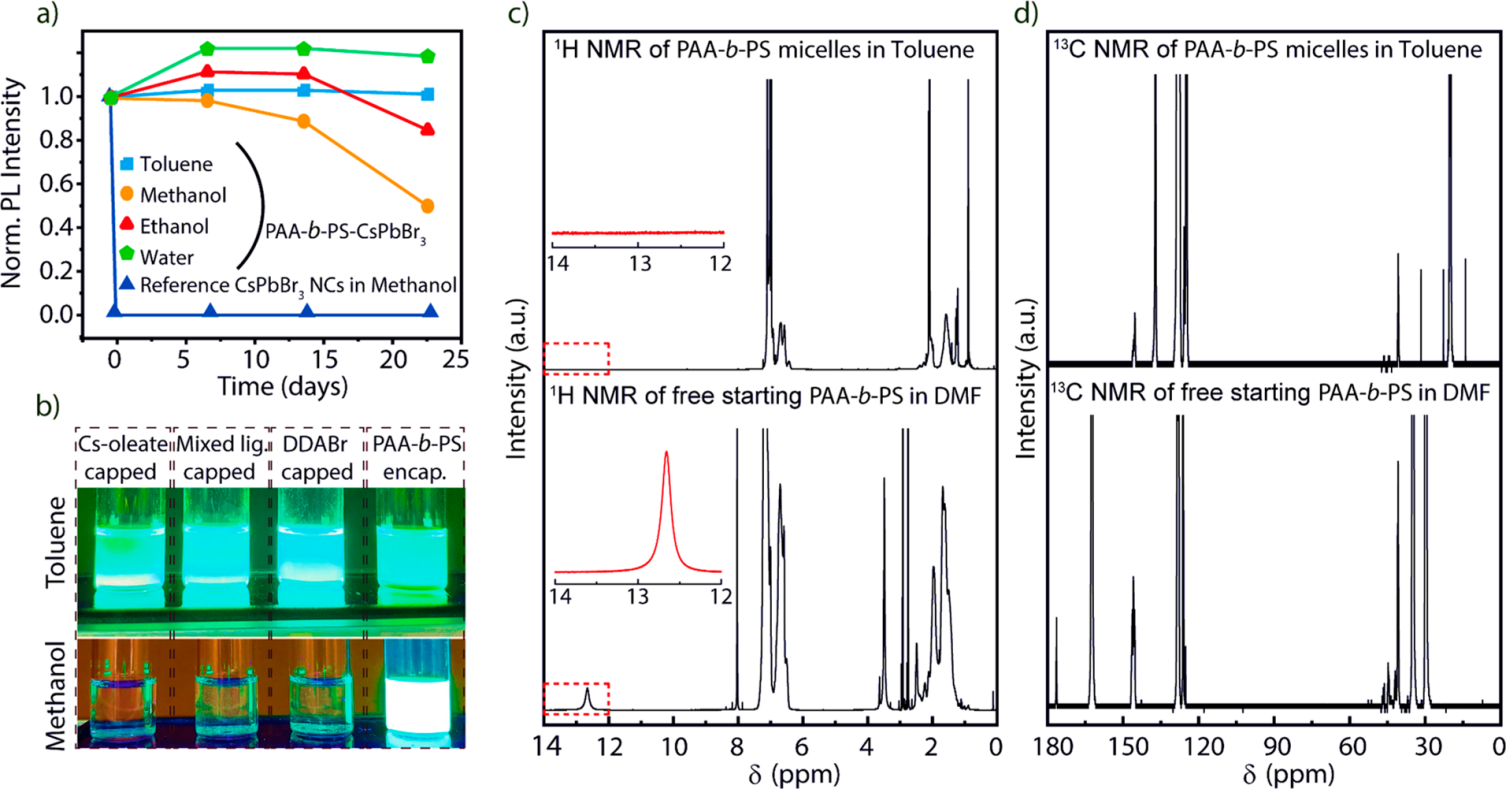
(a) Stability of PAA-*b*-PS-encapsulated CsPbBr_3_ NCs in different solvents, including toluene, water, ethanol, and methanol in comparison to the “reference” CsPbBr_3_ NCs over 23 days (the PL spectra of the corresponding samples are reported in Figure S10). The reference CsPbBr_3_ NCs used for the stability test were synthesized using oleic acid and oleylamine; hence they were not polymer-encapsulated. (b) Photographs of vials containing CsPbBr_3_NCs capped with different ligands (Cs-oleate,^65^ mixed ligands (oleylammonium oleate/bromide)^6^, DDABr^66^), and PAA-*b*-PS-encapsulated CsPbBr_3_ NCs, in toluene and methanol. Panels (c) and (d) report the ^1^H and ^13^C NMR spectra of the free PAA-*b*-PS polymer and the empty PAA-*b*-PS micelles in toluene-*d*_8_ and DMF-*d*_7_, respectively. The empty polymer micelles were prepared in the same way as the polymer-encapsulated NCs, except for the addition of metal halide salts (ABr and PbBr_2_) and additive molecules in the DMF solution.

We performed detailed high resolution liquid state NMR spectroscopy to assess the structural conformation of the PAA-*b*-PS. The NMR experiments were carried out on the starting free PAA-*b*-PS, empty PAA-*b*-PS micelles, and PAA-*b*-PS-encapsulated NCs. The details on the preparation of all the samples used for NMR experiments are reported in the SI. Heteronuclear Single Quantum Coherence (HSQC) and ^1^H–^13^C Heteronuclear Multiple Bond Correlation (HMBC) NMR analyses on the starting free PAA-*b*-PS and empty PAA-*b*-PS micelles in selective (toluene) and nonselective (DMF) solvents; see Figure 3c,d and Figures S11–13. First, the ^1^H and ^13^C NMR analysis on the starting free PAA-*b*-PS in DMF-*d*_7_ evidenced the signals corresponding to both PAA and PS moieties (see Figure 3c,d bottom panel and Figure S11 of SI). The complete ^1^H and ^13^C peak assignment, as well as the HMBC cross correlation peaks, enabled us to unambiguously identify the diagnostic signal (3) in position α to CO (2) of the carboxylic moiety of the PAA component, which does not overlap with the CHs of the PS moiety (see the NMR spectra in DMF in Figure S11A–D). We then carried out the same set of experiments, under identical conditions, on the empty PAA-*b*-PS micelles sample in a toluene-*d*_8_ dispersion. The detailed analysis of the ^1^H, ^13^C, 2D ^1^H–^13^C, HSQC, and ^1^H–^13^C HMBC NMR revealed that the characteristic signals belonging to the PAA component are not detectable (see Figure 3c,d top panel and Figure S12A–D of the SI). On the other hand, the characteristic peaks belonging to the PS component of the polymer could be clearly identified and properly indexed. Here, the inaccessibility to the solvent (responsible for a reduced mobility) of the PAA component of the PAA-*b*-PS micelle is compatible with a model in which PAA forms a rigid core, while PS is present at the outer shell. We further recorded the ^1^H NMR spectrum of the PAA-*b*-PS-encapsulated NCs in toluene-*d*_8_ and compared it with the ^1^H NMR spectrum of the empty micelles (see Figure S12A,B). The spectrum of the empty PAA-*b*-PS micelles had peak profiles and signal widths at half-height that were identical to those of the micelles with the NCs inside, thereby confirming the similar structure of the polymer micelles in both cases. These measurements overall confirm that PAA forms the core of the micelles encapsulating the perovskite NCs, while PS forms the outer shell (see detailed discussion in the NMR section of the SI).

All the samples presented so far were prepared using 5-aminopentanoic acid (APAc), as this molecule led to stable polymer-encapsulated NCs, but other small molecules were also identified as suitable additives. A detailed description of the tests on the various molecules and related discussion are reported in the SI (see also Figures S14–S18 and Table S4). Typical molecules that were tested include hexan-1-amine, hexanoic acid, 2-aminoethanethiol, 4-aminobutanoic acid, 5-aminopentanoic acid, (3-aminopropyl)phosphonic acid, 1,4-butandioic acid, 2-amino-3-hydroxypropanoic acid, pyrrolidine-2-carboxylic acid, 2-amino-3-methylbutanoic acid, 2-amino-5-(diaminomethylideneamino)pentanoic, 2-aminopentanedioic acid, 2-amino-3-sulfhydrylpropanoic acid, 2-amino-3-(1*H*-imidazol-4-yl)propanoic acid, and 2-aminoterphthalic acid. Upon dispersing in methanol, the samples prepared using additive molecules with one functional group (for example, hexan-1-amine, hexanoic acid and even their mixture) significantly lost their PL. On the other hand, the sample prepared using, for example, 5-aminopentanoic acid, which contains two functional groups (carboxylic and amino), remained bright and stable. The results from our tests indicate that effective molecules should be bifunctional or even trifunctional in nature, with either all acidic (carboxylic, phosphonic) groups or a combination of acidic and at most one basic (such as amino) groups. The details about the PL stability of PAA-*b*-PS-encapsulated FAPbBr_3_ NCs prepared by various additives molecules are summarized in Table S4. The optimal concentration of additive molecule(s) was found to be in the range of 10–15 mM. As a control, when no additive molecules were added, all samples were quickly degraded in methanol (Figures S17 and 18). The mechanism underpinning the increased stability when using certain types of additive molecules is presently not entirely clear. Most likely these molecules partially bind to the surface of the NCs and at the same time they interact with the polymer, ensuring a higher compatibility between the two components. In addition, additive molecules having more than two functional groups might also cross-link the PAA core *via* hydrogen bonding and/or ionic bonding,^67,68^ further improving the stability of the PAA-*b*-PS-encapsulated NCs. The relative fraction of these molecules is, however, so low that they cannot be distinguished by NMR or by other techniques against the much dominant signals from the polymer component.

Next, we tested the fluorescence stability of PAA-*b*-PS-encapsulated APbBr_3_ NCs under high flux photon irradiation. Note that all samples considered for the tests were prepared by blending NCs dispersions in toluene with polystyrene (see the SI for details). PAA-*b*-PS-encapsulated CsPbBr_3_, Cs_0.5_FA_0.5_PbBr_3_, and FAPbBr_3_ NCs samples were prepared using APAc as an additive molecule. The “reference” CsPbBr_3_ NCs prepared *via* a standard colloidal approach^6^ and capped with oleylammonium oleate/bromide^69^ ligand pairs (see the SI) were then blended with polystyrene and tested in parallel under identical conditions. A sketch of the setup used for the stability test is shown in Figure 4a. A known amount of powders of the reference CsPbBr_3_ NCs, PAA-*b*-PS-encapsulated CsPbBr_3_, Cs_0.5_FA_0.5_PbBr_3_, and FAPbBr_3_ NCs were placed between two glass slides and irradiated continuously under a flux of 3.2 W/cm^2^ for 60 min at 445 nm. These high flux irradiation conditions are nearly ten times harsher than the required industrial standards for displays (100–400 mW/cm^2^).^70,71^ The PL spectra of the reference NCs and the PAA-*b*-PS-encapsulated NCs were monitored by an acquisition every 60 s *via* a software interface. The spectra are reported in Figure 4b–e and the drop of PL intensities versus irradiation time are reported in Figure 4f. The reference CsPbBr_3_ sample lost a significant fraction of its PL in the first minute, which further dropped down to 18% of its initial intensity after 60 min of exposure to the laser (i.e., a PL loss of 82%). In comparison, over the same time frame, the PAA-*b*-PS-encapsulated CsPbBr_3_, Cs_0.5_FA_0.5_PbBr_3_, and FAPbBr_3_ NC samples lost 23%, 6%, and 8% of their initial PL intensity, respectively, demonstrating their improved stability.

**Figure 4:**
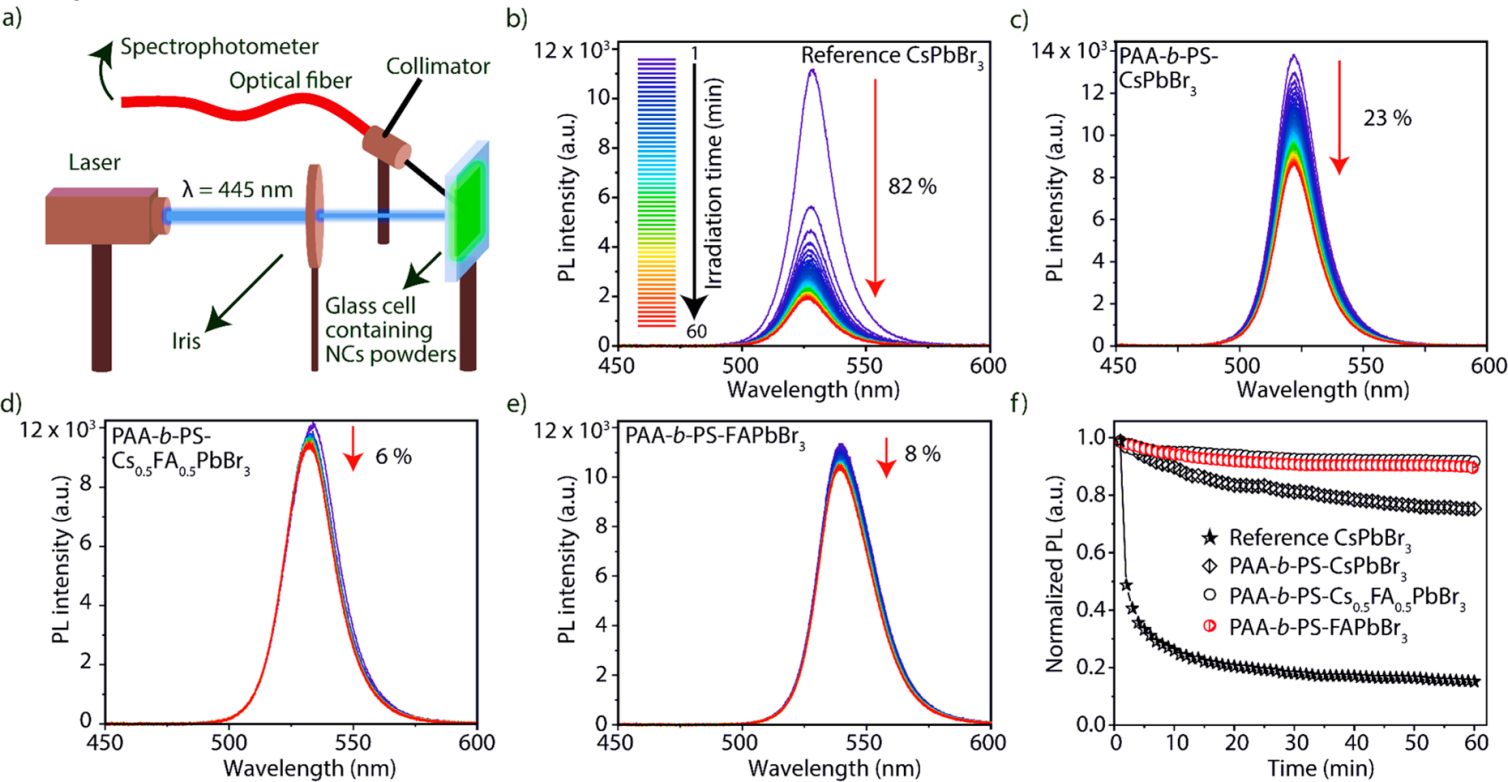
Stability test under high flux irradiation of “reference” CsPbBr_3_ NCs (synthesized using oleic acid and oleylamine; then mixed with polystyrene) versus PAA-*b*-PS-encapsulated NCs. (a) sketch of the experimental setup; (b) evolution of the PL of the reference CsPbBr_3_ NCs, evidencing 82% loss of PL intensity over 60 min of continuous exposure to laser irradiation. Over the same time frame, the PL spectra of the PAA-*b*-PS-encapsulated CsPbBr_3_ (c), Cs_0.5_FA_0.5_PbBr_3_ (d), and FAPbBr_3_ NCs (e) retained 78%, 94%, and 92% of their initial intensity. (f) Summary of all the data sets for panels (b–e) referred to PL intensity (normalized to the initial PL of each sample).

We also tested the effect of an alternative molecule (phenethylammonium, PEABr, which contains only one functional group) on the stability of NCs against high flux radiation. A PAA-*b*-PS-encapsulated Cs_0.5_FA_0.5_PbBr_3_ NCs sample prepared using PEABr was irradiated continuously under a flux of 3.2 W/cm^2^ for 60 min at 445 nm. The sample lost 56% of its initial PL intensity after 60 min of exposure to the laser (Figure S19a), that is, a much bigger loss compared to the sample prepared with APAc and discussed earlier (6%, Figure 4d). Similarly, upon dispersion in methanol, the sample prepared using PEABr evidenced a bigger drop in the PL intensity compared to the sample prepared with APAc (Figure S19b).

Our empirical conclusions are the following: to prepare polymer-encapsulated NCs possessing stable optical features under high flux irradiation conditions and which additionally retain their PL against polar solvents (such as methanol), the requested additive molecules need to have more than one functional group. For example, they need to have two or more acidic (carboxylic, phosphonic) groups, or a combination of acidic and basic (such as amino) groups.

Taking advantage of the enhanced stability and color tunability on demand of these NCs, we prepared multicolored emissive samples. We prepared first the green emitting PAA-*b*-PS-encapsulated CsPbBr_3_ NCs. The red and blue emitting samples were obtained from them by anion exchange in toluene. Once the desired emission color was reached, the polymer shell was “closed” by adding a mixture of methanol and hexane, followed by centrifugation, solution drying, and grounding with a mortar and pestle in a ceramic crucible (see details in the SI). The different powders were then mixed in an appropriate ratio to get a multicolor emitting product that nearly retained the emitting features of the individual components over months of storage under ambient conditions (Figure 5a). Interestingly, the CsPbI_3_ NCs, which are prone to a phase transition to a nonemitting yellow phase and need additional surface or structural engineering for stability,^12,72−74^ were instead stable in the perovskite phase when polymer coated. Inspired by the retention of the emission color and by the excellent stability of the multicolor powders, we then proceeded to prepare a white emitting powder by combining three primary colors (red, green, and blue), fully based on halide perovskite NCs. The A-site cation-dependent color tunability of PAA-*b*-PS-encapsulated APbBr_3_ NCs in the range of 515 to 540 nm gave us access to the pure green emission range (525–535 nm). Therefore, we used PAA-*b*-PS-encapsulated Cs_0.5_FA_0.5_PbBr_3_ NCs samples considering their nearly pure green emission features. The PAA-*b*-PS-encapsulated Cs_0.5_FA_0.5_PbBr_3_ NCs samples were prepared using 4-aminobutanoic acid as an additive molecule while all the other reaction conditions, including concentration of the precursor solutions and of the polymer, were kept the same. TEM images of the pristine green emitting NCs and of the samples after anion exchange reaction (blue and red emitting NCs) are reported in Figure S20. The blue and red samples were derived from the green emitting sample by halide exchange, similarly to the procedure previously described for the PAA-*b*-PS-encapsulated CsPbX_3_ NCs. The PAA-*b*-PS-encapsulated Cs_0.5_FA_0.5_PbX_3_ NCs with desired emission color in toluene dispersion were mixed with poly(methyl methacrylate), known to be an organic glass and suitable for display applications. Then, the polymer shell encapsulating the NCs was closed by adding a methanol/hexane mixture. The precipitate was collected by centrifugation and the dried in a vacuum oven at 40 °C overnight. The dried precipitates were individually ball milled to get the fine powders (see the details in the SI).

**Figure 5:**
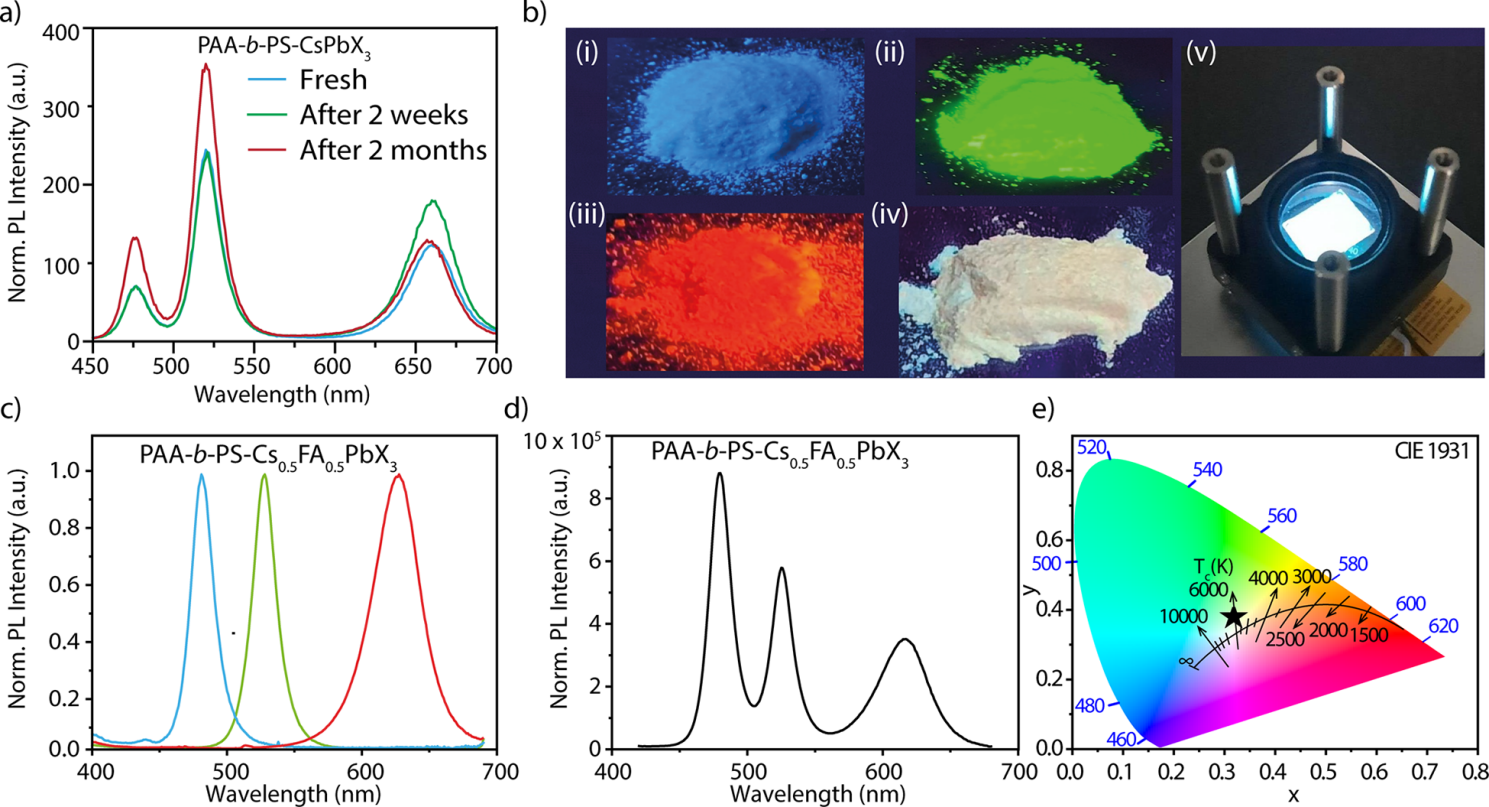
(a) Multicolor fluorescent powders were prepared by mixing the appropriate ratios of PAA-*b*-PS-encapsulated CsPb(BrCl)_3_, CsPbBr_3_, and CsPb(BrI)_3_ NCs powders emitting at 476, 520, and 661 nm, respectively. These powders were prepared from the dried samples, as delivered from the synthesis and, for the blue and red emitting samples, after anion exchange, by grounding with a mortar and pestle in a ceramic crucible. The PL spectra recorded over two of months of aging indicate the retention of the PL peaks position. (b) Photos of the separate blue (i), green (ii), and red (iii) emitting powders of PAA-*b*-PS-encapsulated Cs_0.5_FA_0.5_PbX_3_ NCs and their mixture (iv). These powders were prepared instead by ball milling. All photos were taken while irradiating the samples with a UV lamp. (c) The PL spectra of the separate powders reported in panel (b) (i–iii) with emission centered at 481, 527, and 625 nm for the blue, green, and red samples, respectively. The red, green, and blue powders reported in panel (b) (i–iii) were then blended in appropriate ratios with a water solution of poly(vinyl alcohol) (PVA) to prepare white emitting films for the LED. Panels (d) and (e) show the PL spectra and corresponding CIE coordinates of the obtained white emitting films, respectively. A photograph of a white LED under operational conditions is reported in Panel (b) (v). The device structure for the UV-to-white color converting layer is reported in Figure S23.

The photographs of the powders after ball milling are reported in Figure 5b (i–iii) and the PL spectra recorded from the individual powders are reported in Figure 5c. The PL peak position was centered at 625, 527, and 481 nm for the red, green, and blue NCs powders, respectively. These powders were combined in appropriate ratios to get a white emitting sample. The resulting powder showed nearly white emission under UV-lamp irradiation (Figure 5b (iv), *x* = 0.31 and *y* = 0.38, pure white would be *x* = 0.33 and *y* = 0.33). The PL spectrum recorded on the white emitting powder is reported in Figure S21 and the CIE coordinates are reported in Figure S22. The comparison of the spectral features of the powders before and after mixing and over aging is reported in Figure S21: The individual PL peak positions did not shift after mixing and remained unchanged after 4 weeks of aging under ambient conditions.

The inhibition of halide exchange in the solid matrix further motivated us to fabricate a fully halide perovskite UV-to-white color converting layer combined with a commercial LED. The white emitting films were prepared by blending red, green, and blue powders (reported in Figure 5b (i–iii)) with a water solution of poly(vinyl alcohol) and the sample was drop-cast on quartz substrate (see the details in the SI). The obtained emitting perovskite-PVA film was then placed on a 365 nm LED (maximum power of 1 W) which was used for excitation. A 425 nm long pass filter (Thorlabs) was then placed onto the PVA film to remove the UV excitation light (see Figure S23). The final device featured white emission (Figure 5b (v)) with a conversion ratio of 15% (i.e., 100 mW of 365 nm excitation was converted into 15 mW of white light). The PL spectra and corresponding CIE coordinates of the NC-PVA film used for the white LED are reported in Figure 5d,e.

In conclusion, we have reported an effective *in situ* method to prepare polymer-encapsulated halide perovskite nanocrystals in which we can switch their reactivity toward anion exchange “on demand” by simply changing the polarity of the solvent. The method uses suitable additive molecules that improve the optical stability of the nanocrystals. When protected from anion exchange (for example, when dispersed in polar solvents, or as dried powders), nanocrystals emitting at different colors can be mixed together and the resulting sample preserves the multicolor emission over time. Such color stable samples were further used to prepare fully halide perovskite-based nearly white emitters (and also a white light emitting device based on UV-to-white color converting layer) by simply mixing three primary colors. This unique set of properties, together with a high stability against polar solvents and high-flux irradiation, makes them appealing for a wide range of applications. This method should be easily extendable to other metal halide nanocrystals.

## Supporting Information Available

Video S1: Switchable halide exchange using oleylammonium iodide (AVI)
Video S2: Switchable halide exchange using DDACl (AVI)
Video S3: Triggering halide exchange using DDACl by opening the PS-protecting layer with toluene (AVI)
Video S4: Switchable halide exchange using zinc iodide (MP4)
Materials and methods, extended literature review, table of comparison of key features, TEM, DLS, XRD, and NMR analyses, role of various additives on the stability of perovskite NCs in polar solvents, optical absorption and table of optical properties, PL lifetimes, and PL decay curves, switchable halide exchange reactions, aging tests, synthesis and photographs of PAA-*b*-PS-encapsulated FAPbBr_3_ NCs, additional data on emitting powders and on the white light emitting layer preparation (PDF)

## Supplementary Material

nz1c01232_si_001.avi

nz1c01232_si_002.avi

nz1c01232_si_003.avi

## References

[ref1] Quan L. N.; Rand B. P.; Friend R. H.; Mhaisalkar S. G.; Lee T.-W.; Sargent E. H. Perovskites for next-generation optical sources. Chem. Rev. 2019, 119, 7444–7477. 10.1021/acs.chemrev.9b00107. 31021609

[ref2] Dey A.; Ye J.; De A.; Debroye E.; Ha S. K.; Bladt E.; Kshirsagar A. S.; Wang Z.; Yin J.; Wang Y. State of the Art and Prospects for Halide Perovskite Nanocrystals. ACS Nano 2021, 10.1021/acsnano.0c08903. PMC8482768 34137264

[ref3] Liu X.-K.; Xu W.; Bai S.; Jin Y.; Wang J.; Friend R. H.; Gao F. Metal halide perovskites for light-emitting diodes. Nat. Mater. 2021, 20, 10 10.1038/s41563-020-0784-7. 32929252

[ref4] Gandini M.; Villa I.; Beretta M.; Gotti C.; Imran M.; Carulli F.; Fantuzzi E.; Sassi M.; Zaffalon M.; Brofferio C.; et al. Efficient, fast and reabsorption-free perovskite nanocrystal-based sensitized plastic scintillators. Nat. Nanotechnol. 2020, 15, 462–468. 10.1038/s41565-020-0683-8. 32424340

[ref5] Kovalenko M. V.; Protesescu L.; Bodnarchuk M. I. Properties and potential optoelectronic applications of lead halide perovskite nanocrystals. Science 2017, 358, 745–750. 10.1126/science.aam7093. 29123061

[ref6] Protesescu L.; Yakunin S.; Bodnarchuk M. I.; Krieg F.; Caputo R.; Hendon C. H.; Yang R. X.; Walsh A.; Kovalenko M. V. Nanocrystals of cesium lead halide perovskites (CsPbX_3_, X= Cl, Br, and I): novel optoelectronic materials showing bright emission with wide color gamut. Nano Lett. 2015, 15, 3692–3696. 10.1021/nl5048779. 25633588 PMC4462997

[ref7] Akkerman Q. A.; Rainò G.; Kovalenko M. V.; Manna L. Genesis, challenges and opportunities for colloidal lead halide perovskite nanocrystals. Nat. Mater. 2018, 17, 394–405. 10.1038/s41563-018-0018-4. 29459748

[ref8] Nedelcu G.; Protesescu L.; Yakunin S.; Bodnarchuk M. I.; Grotevent M. J.; Kovalenko M. V. Fast anion-exchange in highly luminescent nanocrystals of cesium lead halide perovskites (CsPbX_3_, X= Cl, Br, I). Nano Lett. 2015, 15, 5635–5640. 10.1021/acs.nanolett.5b02404. 26207728 PMC4538456

[ref9] Shamsi J.; Urban A. S.; Imran M.; De Trizio L.; Manna L. Metal halide perovskite nanocrystals: synthesis, post-synthesis modifications, and their optical properties. Chem. Rev. 2019, 119, 3296–3348. 10.1021/acs.chemrev.8b00644. 30758194 PMC6418875

[ref10] Fu Y.; Zhu H.; Chen J.; Hautzinger M. P.; Zhu X.-Y.; Jin S. Metal halide perovskite nanostructures for optoelectronic applications and the study of physical properties. Nat. Rev. Mater. 2019, 4, 169–188. 10.1038/s41578-019-0080-9.

[ref11] Akkerman Q. A.; D’Innocenzo V.; Accornero S.; Scarpellini A.; Petrozza A.; Prato M.; Manna L. Tuning the optical properties of cesium lead halide perovskite nanocrystals by anion exchange reactions. J. Am. Chem. Soc. 2015, 137, 10276–10281. 10.1021/jacs.5b05602. 26214734 PMC4543997

[ref12] Lu C.-H.; Biesold-McGee G. V.; Liu Y.; Kang Z.; Lin Z. Doping and ion substitution in colloidal metal halide perovskite nanocrystals. Chem. Soc. Rev. 2020, 49, 4953–5007. 10.1039/C9CS00790C. 32538382

[ref13] Imran M.; Ramade J.; Di Stasio F.; De Franco M.; Buha J.; Van Aert S.; Goldoni L.; Lauciello S.; Prato M.; Infante I.; et al. Alloy CsCd_x_Pb_1-x_Br_3_ Perovskite Nanocrystals: The Role of Surface Passivation in Preserving Composition and Blue Emission. Chem. Mater. 2020, 32, 10641–10652. 10.1021/acs.chemmater.0c03825. 33384476 PMC7768894

[ref14] Imran M.; Caligiuri V.; Wang M.; Goldoni L.; Prato M.; Krahne R.; De Trizio L.; Manna L. Benzoyl halides as alternative precursors for the colloidal synthesis of lead-based halide perovskite nanocrystals. J. Am. Chem. Soc. 2018, 140, 2656–2664. 10.1021/jacs.7b13477. 29378131 PMC5908184

[ref15] Yang D.; Li X.; Zeng H. Surface chemistry of all inorganic halide perovskite nanocrystals: passivation mechanism and stability. Adv. Mater. Interfaces 2018, 5, 1701662 10.1002/admi.201701662.

[ref16] Zito J.; Infante I. The Future of Ligand Engineering in Colloidal Semiconductor Nanocrystals. Acc. Chem. Res. 2021, 54, 1555–1564. 10.1021/acs.accounts.0c00765. 33635646 PMC8028043

[ref17] Smock S. R.; Chen Y.; Rossini A. J.; Brutchey R. L. The surface chemistry and structure of colloidal lead halide perovskite nanocrystals. Acc. Chem. Res. 2021, 54, 707–718. 10.1021/acs.accounts.0c00741. 33449626

[ref18] Xue J.; Wang R.; Yang Y. The surface of halide perovskites from nano to bulk. Nat. Rev. Mater. 2020, 5, 809–827. 10.1038/s41578-020-0221-1.

[ref19] Bidikoudi M.; Fresta E.; Costa R. White perovskite based lighting devices. Chem. Commun. 2018, 54, 8150–8169. 10.1039/C8CC03166E. 29951683

[ref20] Chang S.; Bai Z.; Zhong H. In situ fabricated perovskite nanocrystals: a revolution in optical materials. Adv. Opt. Mater. 2018, 6, 1800380 10.1002/adom.201800380.

[ref21] Brennan M. C.; Ruth A.; Kamat P. V.; Kuno M. Photoinduced anion segregation in mixed halide perovskites. Trends Chem. 2020, 2, 282–301. 10.1016/j.trechm.2020.01.010.

[ref22] Li X.; Wu Y.; Zhang S.; Cai B.; Gu Y.; Song J.; Zeng H. CsPbX_3_ quantum dots for lighting and displays: room-temperature synthesis, photoluminescence superiorities, underlying origins and white light-emitting diodes. Adv. Funct. Mater. 2016, 26, 2435–2445. 10.1002/adfm.201600109.

[ref23] Sun C.; Zhang Y.; Ruan C.; Yin C.; Wang X.; Wang Y.; Yu W. W. Efficient and stable white LEDs with silica-coated inorganic perovskite quantum dots. Adv. Mater. 2016, 28, 10088–10094. 10.1002/adma.201603081. 27717018

[ref24] Yoon H. C.; Kang H.; Lee S.; Oh J. H.; Yang H.; Do Y. R. Study of perovskite QD down-converted LEDs and six-color white LEDs for future displays with excellent color performance. ACS Appl. Mater. Interfaces 2016, 8, 18189–18200. 10.1021/acsami.6b05468. 27349270

[ref25] Pathak S.; Sakai N.; Wisnivesky Rocca Rivarola F.; Stranks S. D.; Liu J.; Eperon G. E.; Ducati C.; Wojciechowski K.; Griffiths J. T.; Haghighirad A. A. Perovskite crystals for tunable white light emission. Chem. Mater. 2015, 27, 8066–8075. 10.1021/acs.chemmater.5b03769.

[ref26] Huang H.; Chen B.; Wang Z.; Hung T. F.; Susha A. S.; Zhong H.; Rogach A. L. Water resistant CsPbX_3_ nanocrystals coated with polyhedral oligomeric silsesquioxane and their use as solid state luminophores in all-perovskite white light-emitting devices. Chem. Sci. 2016, 7, 5699–5703. 10.1039/C6SC01758D. 30034709 PMC6022059

[ref27] Yao E. P.; Yang Z.; Meng L.; Sun P.; Dong S.; Yang Y.; Yang Y. High-brightness blue and white leds based on inorganic perovskite nanocrystals and their composites. Adv. Mater. 2017, 29, 1606859 10.1002/adma.201606859. 28394472

[ref28] Huang C.-Y.; Huang S.-J.; Liu M.-H. M. Hybridization of CsPbBr_1. 5_I_1. 5_ perovskite quantum dots with 9, 9-dihexylfluorene co-oligomer for white electroluminescence. Org. Electron. 2017, 44, 6–10. 10.1016/j.orgel.2017.01.042.

[ref29] Chang Y.; Yoon Y. J.; Li G.; Xu E.; Yu S.; Lu C.-H.; Wang Z.; He Y.; Lin C. H.; Wagner B. K. All-inorganic perovskite nanocrystals with a stellar set of stabilities and their use in white light-emitting diodes. ACS Appl. Mater. Interfaces 2018, 10, 37267–37276. 10.1021/acsami.8b13553. 30338971

[ref30] Xuan T.; Huang J.; Liu H.; Lou S.; Cao L.; Gan W.; Liu R.-S.; Wang J. Super-hydrophobic cesium lead halide perovskite quantum dot-polymer composites with high stability and luminescent efficiency for wide color gamut white light-emitting diodes. Chem. Mater. 2019, 31, 1042–1047. 10.1021/acs.chemmater.8b04596.

[ref31] Jang J.; Kim Y. H.; Park S.; Yoo D.; Cho H.; Jang J.; Jeong H. B.; Lee H.; Yuk J. M.; Park C. B. Extremely Stable Luminescent Crosslinked Perovskite Nanoparticles under Harsh Environments over 1.5 Years. Adv. Mater. 2021, 33, 2005255 10.1002/adma.202005255. 33617075

[ref32] Bose R.; Yin J.; Zheng Y.; Yang C.; Gartstein Y. N.; Bakr O. M.; Malko A. V.; Mohammed O. F. Gentle Materials Need Gentle Fabrication: Encapsulation of Perovskites by Gas-Phase Alumina Deposition. J. Phys. Chem. Lett. 2021, 12, 2348–2357. 10.1021/acs.jpclett.0c03729. 33656346

[ref33] Palei M.; Imran M.; Biffi G.; Manna L.; Di Stasio F.; Krahne R. Robustness to High Temperatures of Al_2_O_3_-Coated CsPbBr_3_ Nanocrystal Thin Films with High-Photoluminescence Quantum Yield for Light Emission. ACS Appl. Nano Mater. 2020, 3, 8167–8175. 10.1021/acsanm.0c01525. 33817562 PMC8009476

[ref34] An M. N.; Park S.; Brescia R.; Lutfullin M.; Sinatra L.; Bakr O. M.; De Trizio L.; Manna L. Low-Temperature Molten Salts Synthesis: CsPbBr_3_ Nanocrystals with High Photoluminescence Emission Buried in Mesoporous SiO_2_. ACS Energy Lett. 2021, 6, 900–907. 10.1021/acsenergylett.1c00052. 33842693 PMC8025713

[ref35] Pan S.; Chen Y.; Wang Z.; Harn Y.-W.; Yu J.; Wang A.; Smith M. J.; Li Z.; Tsukruk V. V.; Peng J. Strongly-ligated perovskite quantum dots with precisely controlled dimensions and architectures for white light-emitting diodes. Nano Energy 2020, 77, 105043 10.1016/j.nanoen.2020.105043.

[ref36] Ercan E.; Tsai P.-C.; Chen J.-Y.; Lam J.-Y.; Hsu L.-C.; Chueh C.-C.; Chen W.-C. Stretchable and ambient stable perovskite/polymer luminous hybrid Nanofibers of multicolor fiber mats and their white LED applications. ACS Appl. Mater. Interfaces 2019, 11, 23605–23615. 10.1021/acsami.9b05527. 31252500

[ref37] Zhang H.; Wang X.; Liao Q.; Xu Z.; Li H.; Zheng L.; Fu H. Embedding perovskite nanocrystals into a polymer matrix for tunable luminescence probes in cell imaging. Adv. Funct. Mater. 2017, 27, 1604382 10.1002/adfm.201604382.

[ref38] Zhong Q.; Liu J.; Chen S.; Li P.; Chen J.; Guan W.; Qiu Y.; Xu Y.; Cao M.; Zhang Q. Highly Stable CsPbX_3_/PbSO_4_ Core/Shell Nanocrystals Synthesized by a Simple Post-Treatment Strategy. Adv. Opt. Mater. 2021, 9, 2001763 10.1002/adom.202001763.

[ref39] Ravi V. K.; Scheidt R. A.; Nag A.; Kuno M.; Kamat P. V. To exchange or not to exchange. Suppressing anion exchange in cesium lead halide perovskites with PbSO_4_-oleate capping. ACS Energy Lett. 2018, 3, 1049–1055. 10.1021/acsenergylett.8b00380.

[ref40] Loiudice A.; Strach M.; Saris S.; Chernyshov D.; Buonsanti R. Universal oxide shell growth enables in situ structural studies of perovskite nanocrystals during the anion exchange reaction. J. Am. Chem. Soc. 2019, 141, 8254–8263. 10.1021/jacs.9b02061. 31045360

[ref41] Loiudice A.; Saris S.; Oveisi E.; Alexander D. T.; Buonsanti R. CsPbBr_3_ QD/AlO_x_ inorganic nanocomposites with exceptional stability in water, light, and heat. Angew. Chem., Int. Ed. 2017, 56, 10696–10701. 10.1002/anie.201703703. 28547826

[ref42] Rainò G.; Landuyt A.; Krieg F.; Bernasconi C.; Ochsenbein S. T.; Dirin D. N.; Bodnarchuk M. I.; Kovalenko M. V. Underestimated effect of a polymer matrix on the light emission of single CsPbBr_3_ nanocrystals. Nano Lett. 2019, 19, 3648–3653. 10.1021/acs.nanolett.9b00689. 31117751

[ref43] Baranov D.; Caputo G.; Goldoni L.; Dang Z.; Scarfiello R.; De Trizio L.; Portone A.; Fabbri F.; Camposeo A.; Pisignano D. Transforming colloidal Cs_4_PbBr_6_ nanocrystals with poly (maleic anhydride-alt-1-octadecene) into stable CsPbBr_3_ perovskite emitters through intermediate heterostructures. Chem. Sci. 2020, 11, 3986–3995. 10.1039/D0SC00738B. 32884635 PMC7116022

[ref44] Ushakova E. V.; Cherevkov S. A.; Sokolova A. V.; Li Y.; Azizov R. R.; Baranov M. A.; Kurdyukov D. A.; Yu Stovpiaga E.; Golubev V. G.; Rogach A. L. Stable luminescent composite microspheres based on porous silica with embedded CsPbBr_3_ perovskite nanocrystals. ChemNanoMat 2020, 6, 1080–1085. 10.1002/cnma.202000154.

[ref45] Lu Z.; Li Y.; Qiu W.; Rogach A. L.; Nagl S. Composite films of CsPbBr_3_ perovskite nanocrystals in a hydrophobic fluoropolymer for temperature imaging in digital microfluidics. ACS Appl. Mater. Interfaces 2020, 12, 19805–19812. 10.1021/acsami.0c02128. 32237718

[ref46] Ravi V. K.; Scheidt R. A.; DuBose J.; Kamat P. V. Hierarchical arrays of cesium lead halide perovskite nanocrystals through electrophoretic deposition. J. Am. Chem. Soc. 2018, 140, 8887–8894. 10.1021/jacs.8b04803. 29927589

[ref47] Pang X.; Zhao L.; Han W.; Xin X.; Lin Z. A general and robust strategy for the synthesis of nearly monodisperse colloidal nanocrystals. Nat. Nanotechnol. 2013, 8, 426 10.1038/nnano.2013.85. 23728076

[ref48] Pileni M.-P. The role of soft colloidal templates in controlling the size and shape of inorganic nanocrystals. Nat. Mater. 2003, 2, 145–150. 10.1038/nmat817. 12612669

[ref49] Müllner M.; Lunkenbein T.; Breu J.; Caruso F.; Müller A. H. Template-directed synthesis of silica nanowires and nanotubes from cylindrical core-shell polymer brushes. Chem. Mater. 2012, 24, 1802–1810. 10.1021/cm300312g.

[ref50] Li F.; Wang K.; Deng N.; Xu J.; Yi M.; Xiong B.; Zhu J. Self-Assembly of Polymer End-Tethered Gold Nanorods into Two-Dimensional Arrays with Tunable Tilt Structures. ACS Appl. Mater. Interfaces 2021, 13, 6566–6574. 10.1021/acsami.0c22468. 33522228

[ref51] Meyns M.; Perálvarez M.; Heuer-Jungemann A.; Hertog W.; Ibáñez M.; Nafria R.; Genç A.; Arbiol J.; Kovalenko M. V.; Carreras J. Polymer-enhanced stability of inorganic perovskite nanocrystals and their application in color conversion LEDs. ACS Appl. Mater. Interfaces 2016, 8, 19579–19586. 10.1021/acsami.6b02529. 27454750

[ref52] Vijila C. M.; Kumar K. R.; Jayaraj M. Stokes shift engineered, stable core-shell perovskite nanoparticle-Poly (methyl methacrylate) composites with high photoluminescence quantum yield. Opt. Mater. 2019, 94, 241–248. 10.1016/j.optmat.2019.05.046.

[ref53] Wu H.; Wang S.; Cao F.; Zhou J.; Wu Q.; Wang H.; Li X.; Yin L.; Yang X. Ultrastable inorganic perovskite nanocrystals coated with a thick long-chain polymer for efficient white light-emitting diodes. Chem. Mater. 2019, 31, 1936–1940. 10.1021/acs.chemmater.8b04634.

[ref54] Di D.; Musselman K. P.; Li G.; Sadhanala A.; Ievskaya Y.; Song Q.; Tan Z.-K.; Lai M. L.; MacManus-Driscoll J. L.; Greenham N. C. Size-dependent photon emission from organometal halide perovskite nanocrystals embedded in an organic matrix. J. Phys. Chem. Lett. 2015, 6, 446–450. 10.1021/jz502615e. 25949773 PMC4415888

[ref55] Huang S.; Zhang T.; Jiang C.; Qi R.; Luo C.; Chen Y.; Lin H.; Travas-Sejdic J.; Peng H. Luminescent CH_3_NH_3_PbBr_3_/β-Cyclodextrin Core/Shell Nanodots with Controlled Size and Ultrastability through Host-Guest Interactions. ChemNanoMat 2019, 5, 1311–1316. 10.1002/cnma.201900381.

[ref56] Ko J.; Ma K.; Joung J. F.; Park S.; Bang J. Ligand-Assisted Direct Photolithography of Perovskite Nanocrystals Encapsulated with Multifunctional Polymer Ligands for Stable, Full-Colored, High-Resolution Displays. Nano Lett. 2021, 21, 2288–2295. 10.1021/acs.nanolett.1c00134. 33645994

[ref57] Wang S.; Du L.; Jin Z.; Xin Y.; Mattoussi H. Enhanced Stabilization and Easy Phase Transfer of CsPbBr_3_ Perovskite Quantum Dots Promoted by High-Affinity Polyzwitterionic Ligands. J. Am. Chem. Soc. 2020, 142, 12669–12680. 10.1021/jacs.0c03682. 32588627

[ref58] Raja S. N.; Bekenstein Y.; Koc M. A.; Fischer S.; Zhang D.; Lin L.; Ritchie R. O.; Yang P.; Alivisatos A. P. Encapsulation of perovskite nanocrystals into macroscale polymer matrices: enhanced stability and polarization. ACS Appl. Mater. Interfaces 2016, 8, 35523–35533. 10.1021/acsami.6b09443. 27991752

[ref59] Hou S.; Guo Y.; Tang Y.; Quan Q. Synthesis and stabilization of colloidal perovskite nanocrystals by multidentate polymer micelles. ACS Appl. Mater. Interfaces 2017, 9, 18417–18422. 10.1021/acsami.7b03445. 28524649

[ref60] He Y.; Yoon Y. J.; Harn Y. W.; Biesold-McGee G. V.; Liang S.; Lin C. H.; Tsukruk V. V.; Thadhani N.; Kang Z.; Lin Z. Unconventional route to dual-shelled organolead halide perovskite nanocrystals with controlled dimensions, surface chemistry, and stabilities. Sci. Adv. 2019, 5, eaax4424 10.1126/sciadv.aax4424. 31819900 PMC6884408

[ref61] Hintermayr V. A.; Lampe C.; Löw M.; Roemer J.; Vanderlinden W.; Gramlich M.; Böhm A. X.; Sattler C.; Nickel B.; Lohmüller T. Polymer nanoreactors shield perovskite nanocrystals from degradation. Nano Lett. 2019, 19, 4928–4933. 10.1021/acs.nanolett.9b00982. 31322894 PMC6892581

[ref62] Liu Y.; Wang Z.; Liang S.; Li Z.; Zhang M.; Li H.; Lin Z. Polar Organic Solvent-Tolerant Perovskite Nanocrystals Permanently Ligated with Polymer Hairs via Star-like Molecular Bottlebrush Trilobe Nanoreactors. Nano Lett. 2019, 19, 9019–9028. 10.1021/acs.nanolett.9b04047. 31692361

[ref63] Mai Y.; Eisenberg A. Self-assembly of block copolymers. Chem. Soc. Rev. 2012, 41, 5969–5985. 10.1039/c2cs35115c. 22776960

[ref64] Zhang T.; Li G.; Chang Y.; Wang X.; Zhang B.; Mou H.; Jiang Y. Full-spectra hyperfluorescence cesium lead halide perovskite nanocrystals obtained by efficient halogen anion exchange using zinc halogenide salts. CrystEngComm 2017, 19, 1165–1171. 10.1039/C6CE02314B.

[ref65] Imran M.; Ijaz P.; Baranov D.; Goldoni L.; Petralanda U.; Akkerman Q.; Abdelhady A. L.; Prato M.; Bianchini P.; Infante I. Shape-pure, nearly monodispersed CsPbBr_3_ nanocubes prepared using secondary aliphatic amines. Nano Lett. 2018, 18, 7822–7831. 10.1021/acs.nanolett.8b03598. 30383965 PMC6428374

[ref66] Imran M.; Ijaz P.; Goldoni L.; Maggioni D.; Petralanda U.; Prato M.; Almeida G.; Infante I.; Manna L. Simultaneous cationic and anionic ligand exchange for colloidally stable CsPbBr_3_ nanocrystals. ACS Energy Lett. 2019, 4, 819–824. 10.1021/acsenergylett.9b00140.

[ref67] Hu W.; Wang Z.; Xiao Y.; Zhang S.; Wang J. Advances in crosslinking strategies of biomedical hydrogels. Biomater. Sci. 2019, 7, 843–855. 10.1039/C8BM01246F. 30648168

[ref68] Xu J.; Liu X.; Ren X.; Gao G. The role of chemical and physical crosslinking in different deformation stages of hybrid hydrogels. Eur. Polym. J. 2018, 100, 86–95. 10.1016/j.eurpolymj.2018.01.020.

[ref69] De Roo J.; Ibáñez M.; Geiregat P.; Nedelcu G.; Walravens W.; Maes J.; Martins J. C.; Van Driessche I.; Kovalenko M. V.; Hens Z. Highly dynamic ligand binding and light absorption coefficient of cesium lead bromide perovskite nanocrystals. ACS Nano 2016, 10, 2071–2081. 10.1021/acsnano.5b06295. 26786064

[ref70] Sinatra L.; Lutfullin M.; Lentijo-Mozo S.; Bakr O. M. 16–5: Late-News Paper: High Flux Stable Perovskite Quantum Dots-Polymer Composite for Down-Converting Applications. Dig. Tech. Pap. - Soc. Inf. Disp. Int. Symp. 2020, 51, 222–223. 10.1002/sdtp.13843.

[ref71] Yang D.; Li X.; Zhou W.; Zhang S.; Meng C.; Wu Y.; Wang Y.; Zeng H. CsPbBr_3_ quantum dots 2.0: Benzenesulfonic acid equivalent ligand awakens complete purification. Adv. Mater. 2019, 31, 1900767 10.1002/adma.201900767. 31172615

[ref72] Pan J.; Shang Y.; Yin J.; De Bastiani M.; Peng W.; Dursun I.; Sinatra L.; El-Zohry A. M.; Hedhili M. N.; Emwas A.-H. Bidentate ligand-passivated CsPbI3 perovskite nanocrystals for stable near-unity photoluminescence quantum yield and efficient red light-emitting diodes. J. Am. Chem. Soc. 2018, 140, 562–565. 10.1021/jacs.7b10647. 29249159

[ref73] Wang S.; Bi C.; Portniagin A.; Yuan J.; Ning J.; Xiao X.; Zhang X.; Li Y. Y.; Kershaw S. V.; Tian J. CsPbI_3_/PbSe Heterostructured Nanocrystals for High-Efficiency Solar Cells. ACS Energy Lett. 2020, 5, 2401–2410. 10.1021/acsenergylett.0c01222.

[ref74] Imran M.; Peng L.; Pianetti A.; Pinchetti V.; Ramade J.; Zito J.; Di Stasio F.; Buha J.; Toso S.; Song J.; et al. Halide Perovskite-Lead Chalcohalide Nanocrystal Heterostructures. J. Am. Chem. Soc. 2021, 143, 1435–1446. 10.1021/jacs.0c10916. 33440926 PMC7844828

