# Supplementary material for: Switchable Anion Exchange in Polymer-Encapsulated APbX3 Nanocrystals Delivers Stable All-Perovskite White Emitters
Source: ACS Energy Lett. 2021 Jul 22;6(8):2844–53. doi: 10.1021/acsenergylett.1c01232 (PMC8369489; doi:10.1021/acsenergylett.1c01232)
Supplement: Supplementary file 5 — nz1c01232_si_005.pdf [file nz1c01232_si_005.pdf]

Supporting information for

# Switchable Anion Exchange in Polymer-Encapsulated $APbX_3$ Nanocrystals Delivers Stable All-Perovskite White Emitters

Muhammad Imran[a‡*], Binh T. Mai[b‡], Luca Goldoni[c], Matilde Cirignano[d, e], Houman Bahmani Jalali[d], Francesco Di Stasio[d], Teresa Pellegrino[b*], Liberato Manna[a*]

[a] Nanochemistry, Istituto Italiano di Tecnologia, Via Morego 30, 16163 Genova, Italy

[b] Nanomaterials for Biomedical Applications, Istituto Italiano di Tecnologia, Via Morego 30, 16163 Genova, Italy

[c] Analytical Chemistry Lab, Istituto Italiano di Tecnologia, Via Morego 30, 16163 Genova, Italy

[d] Photonic Nanomaterials, Istituto Italiano di Tecnologia, Via Morego 30, 16163 Genova, Italy

[e] Dipartimento di Chimica e Chimica Industriale, Università degli Studi di Genova, Via Dodecaneso 31, 16146 Genova, Italy

## Contents

Materials and Methods .......... 2

Extended literature review on polymer encapsulated lead halide pervoskite NCs .......... 5

TEM, DLS, XRD and optical analysis .......... 7

Additional data on switchable anion exchange .......... 12

Ageing tests .......... 13

Detailed NMR analysis .......... 13

Stability tests using various additive molecules .......... 18

Additional data on emitting powders and on the white light emitting layer preparation .......... 23

References .......... 25

## Materials and Methods

**Chemicals.** Poly(acrylic acid)-*block*-poly(styrene) (PAA-*b*-PS) (Mw = 33000 g.mol-1, 20% PAA), Lead bromide ($PbBr_2$ ,99%), zinc iodide ($ZnI_2$, 98%) cesium bromide (CsBr, 99%), formamidinium bromide (FABr, 98%) and all additive molecules (Hexan-1-amine, Hexanoic acid, 2-aminoethanethiol, 4-aminobutanoic acid, 5-aminopentanoic acid, (3-aminopropyl)phosphonic acid, 1,4-butandioic acid, 2-amino-3-hydroxypropanoic acid, Pyrrolidine-2-carboxylic acid, 2-amino-3-methylbutanoic acid, 2-amino-5-(diaminomethylideneamino)pentanoic, 2-Aminopentanedioic acid, -2-Amino-3-sulfhydrylpropanoic acid, 2-Amino-3-(1H-imidazol-4-yl)propanoic acid, 2-aminotertphthalic acid), poly(vinyl alcohol) (PVA, Mw 85,000-124,000), Oleylamine (OLAM, 98%), Polystyrene (PS, Mw 35,000) and Iodine ($I_2$, 99.99%) were purchased from Sigma-Aldrich. Didodecyldimethylammonium Chloride (DDACl, 98%) was purchased from TCI. All the chemicals were used as received. All the solvents (hexane, toluene, dimethylformamide (DMF) were also purchased from Sigma-Aldrich and used without further purification.

**Synthesis of Polymer-encapsulated $APbBr_3$ Nanocrystals.** Metal bromide salts (CsBr, FABr and $PbBr_2$: 10 mM each) additive molecules (for example 5-aminopentanoic acid, APAc, the concentration of the additive molecules was kept the same - 17 mM - in all experiments) and polymer (PAA-*b*-PS, 110 mg/mL) were first separately dissolved in DMF. Then, a mixture of ABr (100 µL), $PbBr_2$ (100 µL), APAc (4 µL) and PAA-*b*-PS (75 µL) precursor's solutions in DMF was injected dropwise into a 20 mL vial containing toluene (8 mL) under vigorous stirring at room temperature in air (see figure 1). The color of the solution turned green immediately, indicating the formation of perovskite NCs. The mixed cation $Cs_{0.5}FA_{0.5}PbBr_3$ NCs were prepared by using 1:1 molar ratios of CsBr and FABr precursors directly in the synthesis. After about 20 seconds, the reaction was quenched by adding excess of hexane and the NCs were collected by centrifugation. Then, the supernatant was discarded and the precipitate was re-dispersed in toluene followed by another round of centrifugation and re-dispersion. The toluene dispersion of the NCs was centrifuged once again (at 6000 rpm for 10 min), the colloidally unstable fraction was discarded and the supernatant was collected for further uses.

**Halide exchange reactions: All the reactions were performed under ambient conditions.** To tune the emission color from green to blue, green emitting polymer-encapsulated $CsPbBr_3$ NCs dispersions in toluene were mixed with a DDACl solution (pre-dissolved in toluene, 25 mM concentration) under continuous stirring. To prepare the red emitting NCs, oleylammonium iodide (OLAM-I) (prepared separately by reacting molecular iodine with oleylamine, 0.4 M $I^-$)[1] was used instead. We observed that the oleylammonium iodide is weakly soluble in methanol. To further verify the switchable behavior of the iodide exchange reaction in our polymer encapsulated NCs, we choose $ZnI_2$ salt as a source of iodine ions. Notably, $ZnI_2$ is high soluble in methanol and has been used for halide exchange reactions in toluene dispersions of halide exchange reactions in toluene dispersions of perovskite NCs.[2] Briefly, $ZnI_2$ was directly added in toluene and methanol dispersions of PAA-*b*-PS encapsulated green emitting NCs. In the case of the methanol dispersion, the color of the starting NCs was preserved, despite the addition of a large excess of $ZnI_2$. On the other hand, in the toluene dispersion of the NCs, the color of the sample quickly changed from green to red upon addition of $ZnI_2$.

**Preparation of the white emitting powder.** The PAA-*b*-PS encapsulated $Cs_{0.5}FA_{0.5}PbBr_3$ NCs were prepared first using aminobutyric acid as an additive molecule and subsequently subjected to halide exchanged reactions for the preparation of other compositions. Briefly, the toluene dispersion of PAA-*b*-PS-$Cs_{0.5}FA_{0.5}PbBr_3$ NCs were treated with DDACl and OLAM-I to obtain blue and red emitting samples respectively. Once the desired emission color for the blue (480 nm) and red (625 nm) emitting samples was achieved, hexane in excess was added to the NCs dispersion (with volume ratio of 1:5, toluene to hexane) and the resulting mixture was centrifuged at 6000 rpm for 5 minutes. Thereafter, the supernatant was discarded and the precipitate was re dispersed in toluene. Then, PAA-*b*-PS-$Cs_{0.5}FA_{0.5}PbX_3$ NCs with desired emission color in toluene dispersion were separately mixed with poly(methyl methacrylate) pre-dissolved in toluene (50 mg/mL). Finally, the polymer shell of PAA-b-PS micelles

encapsulating the NCs was "closed" by adding a mixture of methanol and hexane (with a volume ratio of 1:5) to the toluene dispersion of the PAA-*b*-PS-$Cs_{0.5}FA_{0.5}PbX_3$ (X=Br and Br/Cl) NCs, while only hexane was added in case of PAA-*b*-PS-$Cs_{0.5}FA_{0.5}Pb(BrI)_3$ NCs. The resulting mixture was centrifuged again at 6000 rpm for 5 minutes and the supernatant was discarded. The precipitate was dried in a vacuum oven at 40 °C overnight. The obtained powders were individually ground with a ball mill (SPEX SamplePrep 8000M MIXER/MILL). The green and blue emitting samples were ball milled for 5 minutes, while red emitting sample for 2 minutes. The obtained fine powders were then mixed in an appropriate ratio to get a white emitting NCs powder.

**White LED fabrication.** The white emitting samples were prepared by mixing red, green and blue emitting powders of PAA-*b*-PS encapsulated $Cs_{0.5}FA_{0.5}PbX_3$ (X=Br and Br/Cl) NCs in an approximate ratio of 1:2:3 in weight (green:blue:red) was added to the water solution of polyvinyl alcohol (PVA, 210 mg/mL). Thereafter, the sample was drop-cast on a quartz substrate and subsequently dried for 15 min at 65°C. Then, white emitting film was placed on a 365 nm LED (maximum power of 1 W) which was used for excitation. A 425 nm long pass filter (Thorlabs) was then placed onto the PVA film to remove the UV excitation light, see the device structure in Figure S23.

**Morphological characterization**. TEM images of the NC samples were acquired with a JEOL-1100 transmission electron microscope operating at an acceleration voltage of 100 kV. Samples were prepared by drop casting diluted solutions of NCs onto carbon film-coated 200 mesh copper grids.

**Structural characterization**. Structural analysis was performed on a PANanalytical Empyrean X-ray diffractometer, equipped with a 1.8 kW CuK$\alpha$ ceramic X-ray tube, operating at 45 kV and 40 mA, and a PIXcel3D 2×2 area detector. A colloidal dispersion of corresponding NCs was drop-cast on a zero-diffraction silicon substrate. All the diffraction patterns reported in this work were collected at room temperature under ambient conditions using parallel beam geometry and symmetric reflection mode. Post-acquisition XRPD data analysis was carried out using the HighScore 4.1 software from PANalytical.

**Spectroscopic measurements.** Optical absorption spectra were recorded using a Varian Cary 300 UV-VIS absorption spectrophotometer. The PL spectra were measured on a Varian Cary Eclipse spectrophotometer using an excitation wavelength ($\lambda_{ex}$) of 350 nm for all the samples. Samples were prepared by diluting NC solutions in toluene, in quartz cuvettes with a path length of 1 cm. Absolute Photoluminescence quantum yields of NC samples were measured using an Edinburgh FLS900 fluorescence spectrometer equipped with a Xenon lamp, a monochromator for steady-state PL excitation, and a time-correlated single photon counting unit coupled with a pulsed laser diode ($\lambda_{ex}$ = 405 nm, pulse width = 50 ps) for time-resolved PL. The PLQY was measured using a calibrated integrating sphere ($\lambda_{ex}$ = 350 nm for all samples). For the PLQY measurements, all the NC dispersions were diluted to an optical density of 0.1 ±0.02 at the corresponding excitation wavelength in order to minimize the amount of fluorophore being reabsorbed.

**Sample preparation for high flux irradiation experiments.** PAA-*b*-PS encapsulated $CsPbBr_3$, $Cs_{0.5}FA_{0.5}PbBr_3$ and $FAPbBr_3$ NCs samples were prepared using AVAc as an additive molecule. The "reference" $CsPbBr_3$ NCs were prepared using hot injection method developed by Protesescu et al.[3] The toluene dispersions of all samples including the reference sample with the concentration 10-12 mg/mL were mixed with a polystyrene solution (30 mg/mL in toluene). The resulting mixture was precipitated by adding 12 mL of hexane, followed by overnight drying in vacuum oven at 40 °C. Then, the powder was grounded by using a mortar and pestle set and was then placed inside a glass cell.

**Laser irritation stability tests.** Stability tests were carried out using a continuous wave laser diode for excitation (λex = 445 nm, Oxxius LBX-445-650-HPE-PP, max power 715 mW) coupled with an iris to reduce the circular excitation spot to a diameter of 1mm (spot size was determined with Thorlabs BP209-VIS/M beam profiler). The excitation power was measured after the iris using a Thorlabs PM100D Digital Optical Power meter interfaced with a Thorlabs S121C photodiode. The emission was monitored via a collimator/optical fiber/filter

holder/optical fiber assembly coupled with an Ocean Optics HR4000 spectrometer. The collection optics was placed at 45° with the respect to the excitation beam and a long pass filter (λcut-off = 450 nm, Thorlabs FEL0450) was placed in the fiber coupled filter holder. The emission was collected every 60 seconds using the Ocean View software.

**DLS measurements**. The hydrodynamic sizes of the particles were measured by Dynamic Light Scattering (DLS) using a Malvern Instruments Zetasizernano series instrument. Prior to each reading, the sample was left for 1 min to equilibrate and triplicate measurements were done for each sample.

**NMR measurements**. NMR experiments were performed at 298 K on a Bruker AvanceIII 600 MHz spectrometer equipped with 5 mm QCI cryoprobe with z shielded pulsed-field gradient coil. The free polymer sample was prepared by dissolving 8.25 mg of PAA-b-PS powder in 1mL of DMF-d7. The PAA-b-PS micelles sample was prepared by dissolving the same amount of polymer into DMF followed by drop-wise addition of the polymer solution into toluene to induce the micelle formation. Thereafter, the micelles were collected by adding hexane in access to the crude solution followed by centrifugation at 5000 rpm for 5 minutes. The supernatant was discarded and the dried precipitate was redispersed in d-8 toluene. For the liquid state NMR measurements, the samples were transferred into 5 mm disposable NMR tubes (Bruker). Before each acquisition, automatic matching and tuning (both on 1H and 13C) and homogeneity were adjusted.

In the *$^{1}$H-NMR experiments*, 128 transients were accumulated after having applied a 90 degree of flip angle excitation pulse, with relaxation delay of 30 s, over a spectral width of 20.55 ppm (offset at 10 ppm).

In the *$^{13}$C NMR spectra* (inverse gated $^{1}$H decoupling), 10752 transients were collected after a 30-degree pulse, with 32768 of digit points, an interpulses delay of 3.5 s, over a spectral width of 240.1 ppm (offset at 100 ppm).

An apodization exponential function equivalent to 0.1 and 15 Hz were applied to the $^{1}$H and $^{13}$C FIDs respectively, before the Fourier transform.

The *$^{1}$H-$^{13}$C HSQC* (multiplicity edited Heteronuclear Single Quantum Coherence, with the selection of $CH_2$ and $CH/CH_3$ in opposite phases, graphically represented in blue and red respectively) was performed with 32 FIDs, 2048 data points, 256 increments, over a spectral width of 15.02 ppm for $^{1}$H and 165.7 ppm for $^{13}$C (transmitter frequency offsets at 7.45 and 74.6 ppm, respectively).

The *$^{1}$H-$^{13}$C HMBC* (Heteronuclear Multiple Bond Correlation) was acquired with 128 FIDs, 2048 data points, 128 increments, over spectral width of 15.02 ppm for $^{1}$H and 222.1 ppm for $^{13}$C (transmitter frequency offsets at 7.48 and 99.8 ppm, respectively).

All spectra were referred to the not deuterated residual solvent peaks, at 7.09 and 129.2 ppm (toluene-$d_7$), and at 8.03 and 163.2 ppm (DMF-$d_6$), for $^{1}$H and $^{13}$C, respectively.

## Extended literature review on polymer encapsulated lead halide pervoskite NCs

Various studies have studies have shown that embedding perovskite NCs in different polymeric matrices significantly improves their stability. Among those, encapsulation of individual MHP NCs is most the appealing and gain signifincat attention of the scientific community in recent past. Hou et al. back in 2017 used polystyrene-*block*-poly-2-vinylpyridine (PS-*b*-P2VP) micelles to prepare PS-*b*-P2VP coated $CsPbX_3$ NCs.[4] This strategy was later adopted by Lohmuller et al. to prepare the PS-*b*-P2VP coated $MAPbX_3$ NCs.[5] In both studies, the nanoreactors were prepared initially by dissolving P4VP-*b*-PS in toluene, followed by the addition of the corresponding halide salts (such as CsX, MAX and $PbX_2$) sequentially. Given a lower solubility of metals salts in toluene, Hou et al. noted that the mixture of nanoreactors and metal halide salts ($PbBr_2$) needed to be kept under continuous stirring for at least two weeks to achieve 0.1 mg/mL loading of the corresponding salt. This however makes the procedure reported by Hou et al. and Lohmuller et al. et al. not only time consuming but also not much useful, as it leads to a very low yield of the final material. In terms of stability, Hou et al. reported that PS-*b*-P2VP capped $CsPbBr_3$ NCs completely lost their PL in a mixture of methanol and toluene (with a volume ratio of 10 to 1) within 2 minutes upon dispersion (see Figure 3 of their manuscript). Lohmuller et al., on the other hand, reported that the PS-*b*-P2VP encapsulated $MAPbBr_3$ NCs have improved stability against moisture, water and weak UV irradiation. The high stability of perovskite NCs against a short chain alcohol such as methanol remains an unachieved goal so far. This is most likely due to the fact that methanol is less hydrophilic and has a lower surface tension compared to water, hence it will have higher tendency to penetrate through the PS protective layer which consequently makes it more detrimental for the perovskite NCs compared even to water. This was indeed observed experimentally on the PS-*b*-P2VP capped perovskite NCs prepared by Hou et al.

Lin et al. recently used a multiarm star-like amphiphilic triblock copolymer, namely poly(4-vinylpyridine)-*block*-poly(tert-butyl acrylate)-*block*-polystyrene (denoted P4VP-*b*-PtBA-*b*-PS), to encapsulate perovskite NCs.[6] In that study, a three-step synthesis was used to prepare $MAPbX_3$ NCs coated with a $SiO_2$ middle layer and PS as the outer shell. Due to the unique molecular structure of the polymer, the micelles are made of 21 chains of triblock copolymer ($1^{st}$ segment of P4VP for $MAPbX_3$ nucleation, 1 middle segment of PtBA to yield PAA upon the thermolysis to bestow the capability to grow a silica shell and last outer segment of PS to improve the stability) linked together through a knot at the end of the P4VP block. Although this approach is elegant, the micelles made of multi-arm star-like amphiphilic triblock copolymers have a rather low density of PS shell (21 chains per micelles) in comparison to the conventional self-assembled micelles made of linear diblock copolymers. This in turn provides a low barrier against a polar environment. Indeed, the authors observed that the growth of the $SiO_2$ shell between the PS layer and the $MAPbX_3$ layer is crucial to achieve a good stability against water. Also in that case, the stability against short chain alcohols was not addressed. Similarly, in another study reported by the same group, Liu et al. used a star-like molecular bottlebrush trilobe, poly(2-hydroxyethyl methacrylate)-*graft*-(poly(acrylic acid)-*block*-partially cross-linked polystyrene (denoted PHEMA-*g*-(PAA-*b*-cPS)) to act as a polymeric nanoreactor.[7] The authors reported that the $CsPbBr_3$ is formed within the PAA block while PS block acts as the protective layer. Owing to its trilobe structure, such polymer will not be able to self-assemble to form a classical micellar structure due to the constraint of the chain. This, in turn, will create some defects in the protective PS layer (not uniform hairy shell), thus allowing the penetration of solvents through the PS shell. The authors indeed reported that polymer coated NCs could withstand 20-30% volume of methanol for less than 20 days. Further increase in the methanol volume (40 % or higher) resulted in a complete loss of PL instantly upon dispersion.

## Table S1: Comparison of key features of the present work with previous reports

| | Material type ($APbX_3$) | Polymer composition | Synthesis conditions | Switchable anion exchange reaction | Stability against polar solvents (Colloidal) | | | High flux irradiation stability | Stable multicolor emitters | Fully perovskite based white LED |
|---|---|---|---|---|---|---|---|---|---|---|
| | | | | | Water | Ethanol | Methanol | | | |
| This study | Cs, FA | PAA-b-PS, linear | single step, RT | Yes | Complete retention of PLafter 23 days (100% water) | 20% of PL lost after 23 days (100% EtOH )) | 50% of PL lost after 23 days (100% MeOH) | Yes (3.2 W/cm$^2$) | Yes | Yes |
| Ref. [4] | Cs | P4VP-b-PS, linear | 2 step, RT | No | Complete loss of PL was observe after 25 hours of dispersion | Complete loss of PL after 40 days (90% EtOH in toluene) | Complete loss of PL upon dispersion (81% methanol in toluene) | N.A. | N.A. | NO |
| Ref. [6] | MA | P4VP-b-PAA-b-PS Multi-arm star like (21 arms per micelles). The growth of SiO2 layer in PAA block is crucial for the stability | 3 step, HT | No | Yes (in film) | N.A. | N.A. | N.A | Yes | NO |
| Ref. [5] | MA | P4VP-b-PS, linear | 2 step, RT | No | Yes (in film) | N.A. | N.A. | N.A. | Yes | NO |
| Ref. [7] | Cs | PAA-b-PS-grafted PHEMA, trilobe | single step, RT | No | Nearly stable against10 % water | N.A | Not stable in complete methanol dispersion | N.A | N.A | NO |

## TEM, DLS, XRD and optical analysis

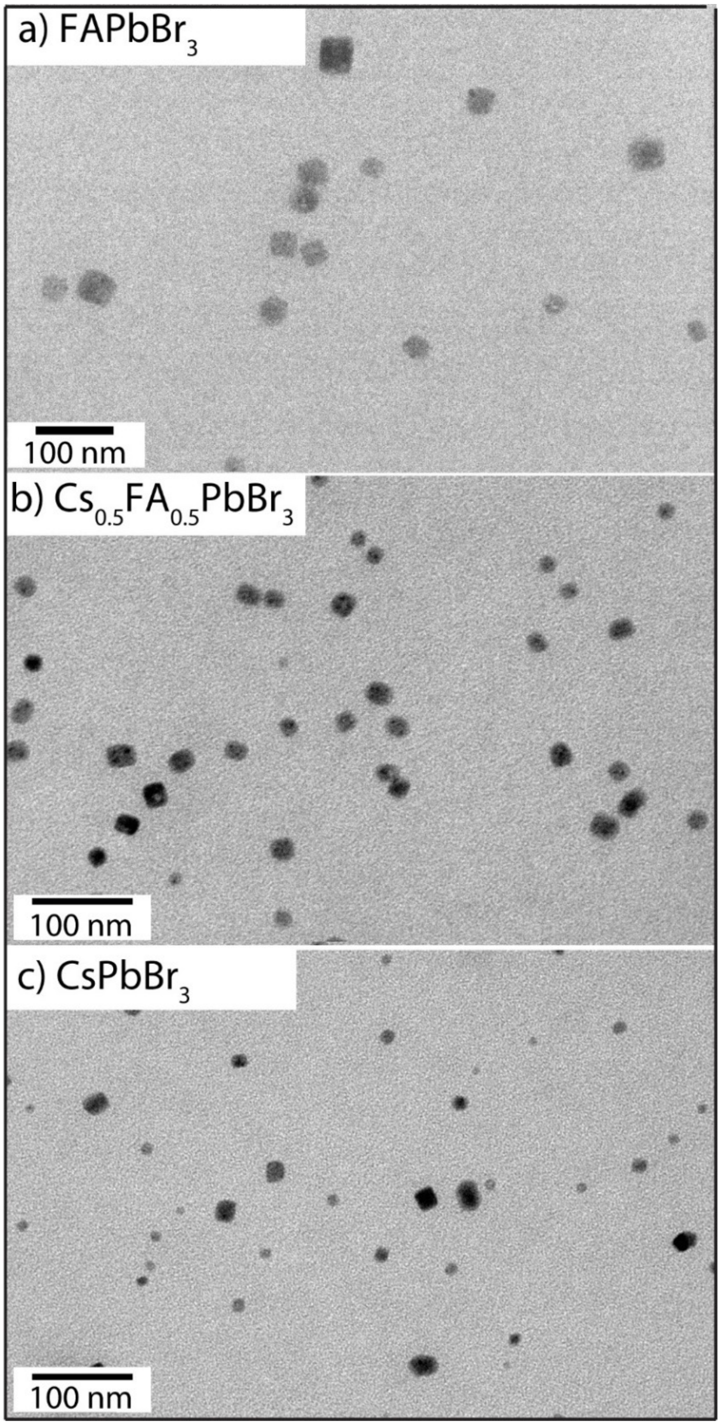


**Figure S1.** TEM images of PAA-*b*-PS encapsulated $CsPbBr_3$, $Cs_{0.5}FA_{0.5}PbBr_3$ and $FAPbBr_3$ NCs samples deposited from toluene dispersions.

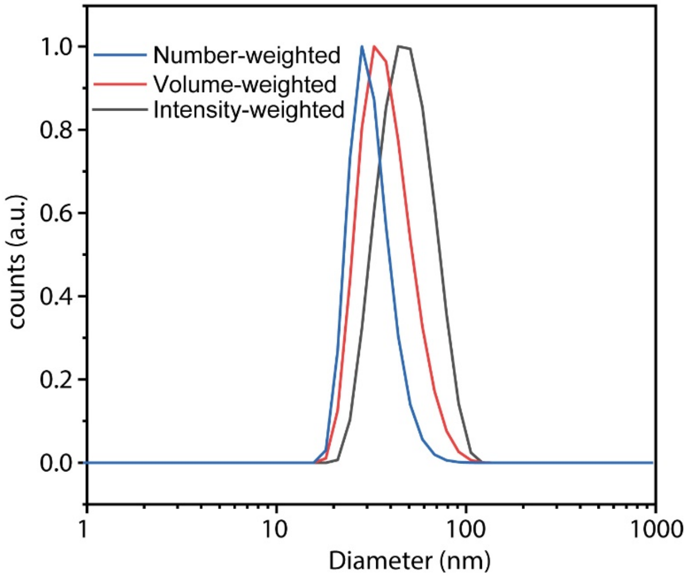


**Figure S2.** Dynamic light scattering (DLS) traces of PAA-*b*-PS dispersions in toluene.

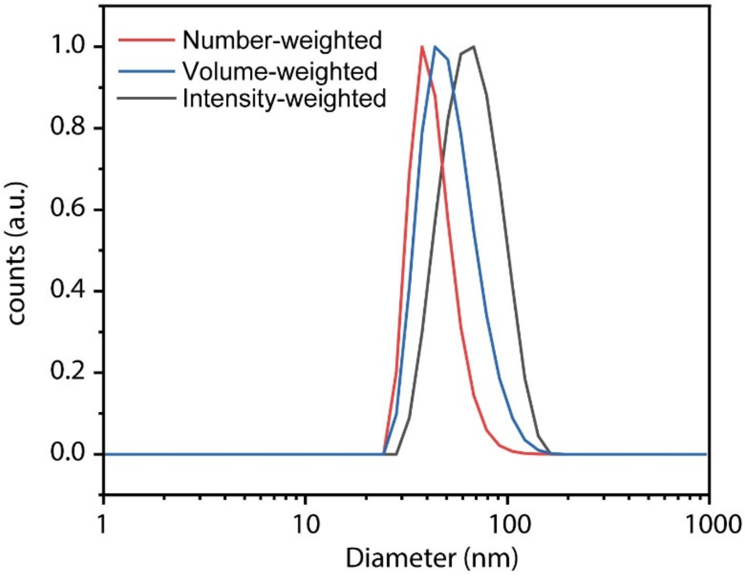


**Figure S3.** DLS traces of PAA-*b*-PS encapsulated $CsPbBr_3$ NCs dispersions in toluene.

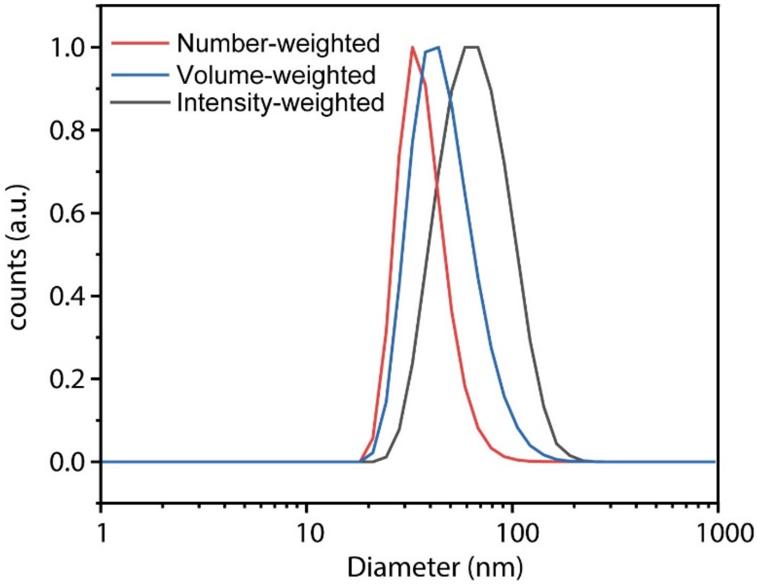


**Figure S4** DLS traces of PAA-*b*-PS encapsulated $Cs_{0.5}FA_{0.5}PbBr_3$ NCs dispersions in toluene.

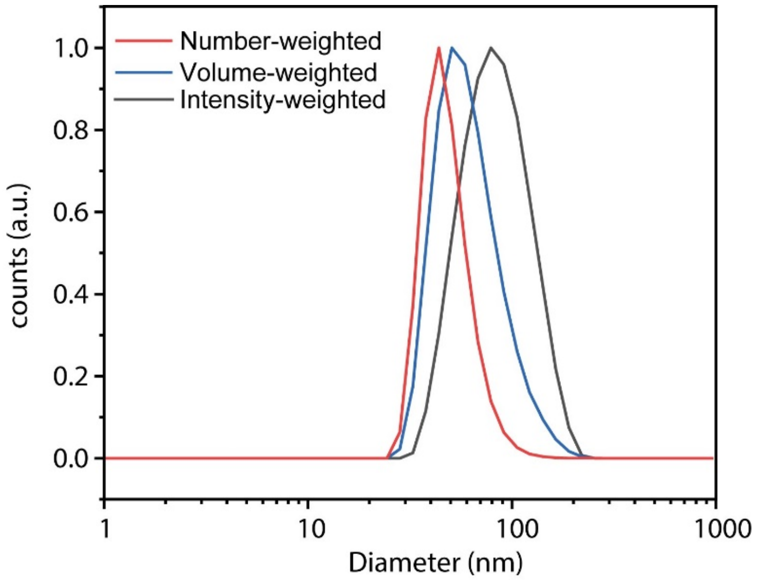


**Figure S5.** DLS traces of PAA-*b*-PS encapsulated $FAPbBr_3$ NCs dispersions in toluene.

**Table S2.** Hydrodynamic diameters ($d_H$) weighted by intensity, volume and number along with polydispersity index (PDI) of free micelles and PAA-*b*-PS encapsulated $CsPbBr_3$, $Cs_{0.5}FA_{0.5}PbBr_3$, $FAPbBr_3$ NCs.

| Sample | $d_H$ (intensity) (nm) | $d_H$ (volume) (nm) | $d_H$ (number) (nm) | PDI |
|---|---|---|---|---|
| PAA-*b*-PS free micelles | 50 ± 1 | 39 ± 1 | 32 ± 1 | 0.10 ± 0.01 |
| PAA-*b*-PS-$CsPbBr_3$ | 69 ± 1 | 53 ± 1 | 43 ± 1 | 0.08 ± 0.01 |
| PAA-*b*-PS-$Cs_{0.5}FA_{0.5}PbBr_3$ | 69 ± 3 | 49 ± 2 | 38 ± 3 | 0.13 ± 0.03 |
| PAA-*b*-PS-$FAPbBr_3$ | 86 ± 1 | 65 ± 1 | 49 ± 1 | 0.06 ± 0.01 |

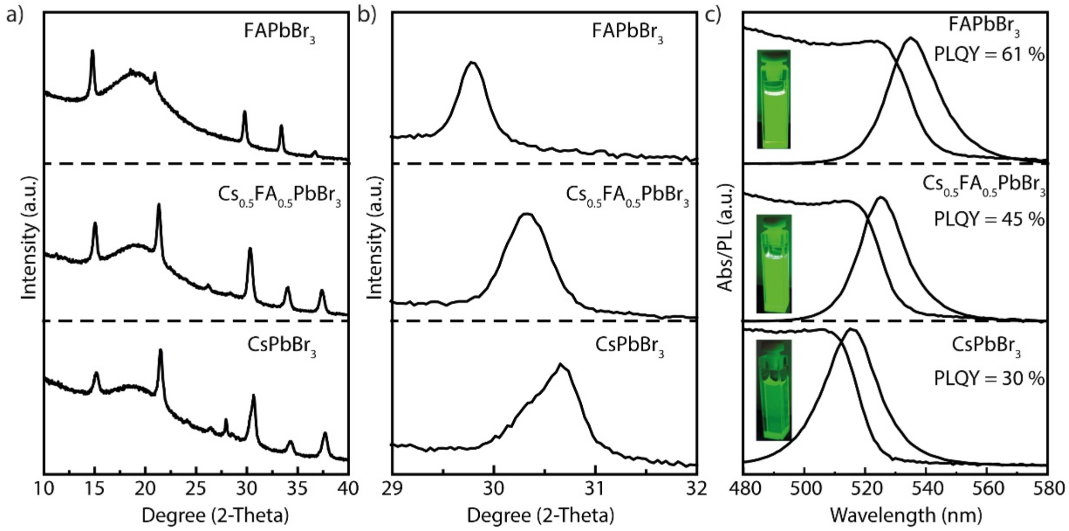


**Figure S6.** (a) XRD patterns of PAA-*b*-PS encapsulated $CsPbBr_3$, $Cs_{0.5}FA_{0.5}PbBr_3$ and $FAPbBr_3$ NCs. The small hump at 18° (2θ) present in all three samples can be ascribed to the presence of the polymer. (b) Magnified view of the XRD patterns reported in (a), in the 29-32° 2θ range (b). (c) Optical absorption and PL spectra recorded on toluene dispersions of the various samples, along with photos of the same samples recorded under the irradiation of a UV lamp. The extended range of absorption and PL spectra of these sample is reported in Figure S7.

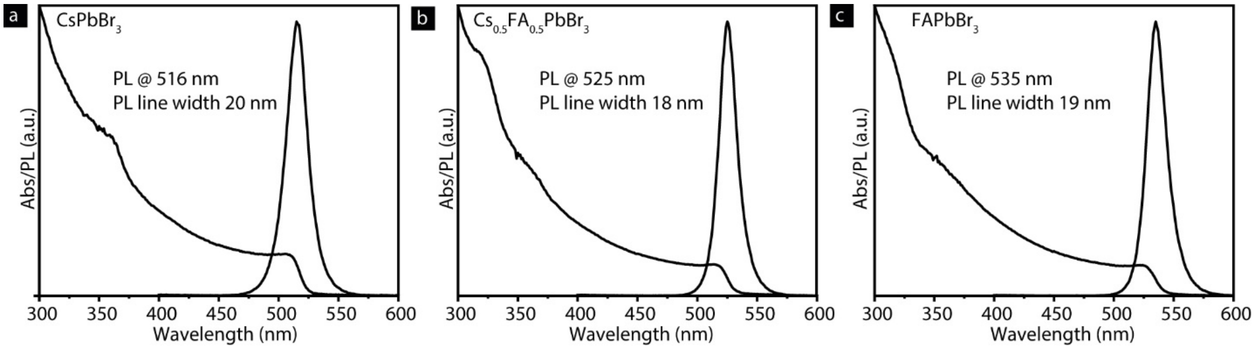


**Figure S7**. Optical absorption and PL spectra of PAA-b-PS encapsulated (a) $CsPbBr_3$, (b) $Cs_{0.5}FA_{0.5}PbBr_3$ and (c) $FAPbBr_3$ NCs dispersions in toluene.

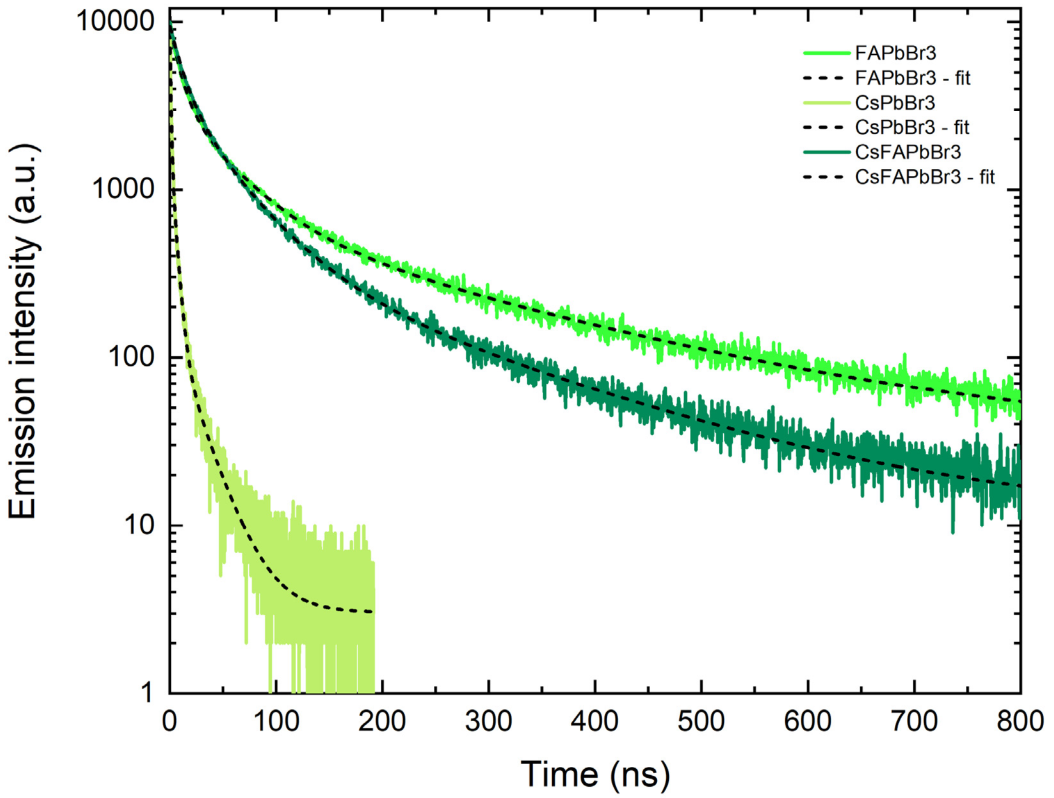


**Figure S8**. PL lifetimes of the PAA-b-PS encapsulated NCs reported in Figure S1 and S6. The measurements were performed in toluene dispersions. The PL decays were fitted with the following expression: Fit = A + B1*exp(-t/T1) + B2*exp(-t/T2) + B3*exp(-t/T3) (values reported in Table S1).

**Table S2:** PL lifetime data extracted from the fittings of plots reported in Figure S8 of PAA-b-PS encapsulated NCs. The reported amplitude and intensity weighted average lifetime were calculated using the following expressions:

Intensity Average lifetime = (B1*(T1)^2+B2*(T2)^2+B3*(T3)^2)/(B1*T1+B2*T2+B3*T3), Amplitude Average lifetime = (B1*T1+B2*T2+B3*T3)/(B1+B2+B3).

| **Sample name** | **Lifetime (ns)** | | **Intensity (a.u.)** | **Rel. Contribution (%)** | **Intensity weighted Average lifetime (ns)** | **Amplitude weighted Average lifetime (ns)** |
|---|---|---|---|---|---|---|
| **$CsPbBr_3$** | | | | | | |
| **T1** | 0.8 | **B1** | 9884 | 41.58 | 6 | 1.64 |
| **T2** | 4.2 | **B2** | 1896 | 41.21 | | |
| **T3** | 22.8 | **B3** | 147 | 17.21 | | |
| **$Cs_{0.5}FA_{0.5}PbBr_3$** | | | | | | |
| **T1** | 11.8 | **B1** | 5582 | 21.24 | 75.9 | 32.2 |
| **T2** | 44.5 | **B2** | 3547 | 50.99 | | |
| **T3** | 183 | **B3** | 468 | 27.77 | | |
| **$FAPbBr_3$** | | | | | | |
| **T1** | 9.1 | **B1** | 6126 | 15.86 | 124 | 35.9 |
| **T2** | 46 | B2 | 2944 | 38.63 | | |
| **T3** | 231 | B3 | 690 | 45.51 | | |

## Additional data on switchable anion exchange

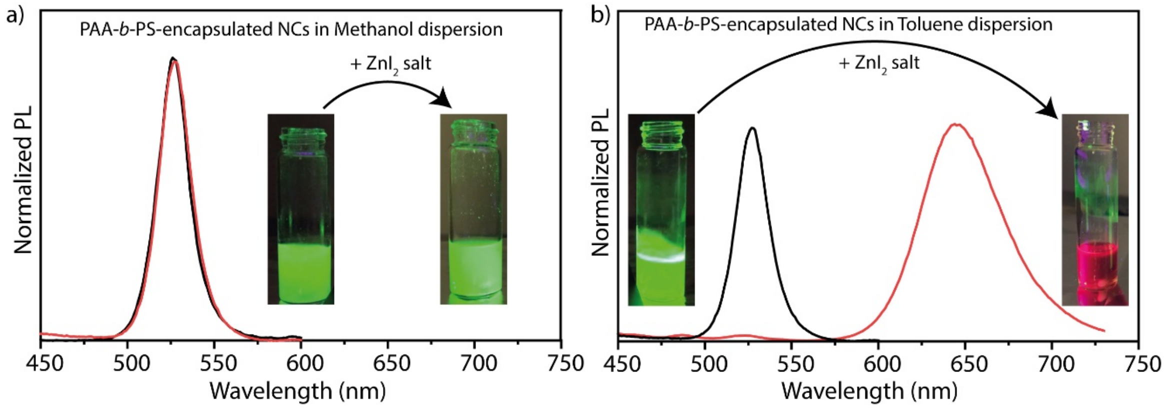


**Figure S9.** Switchable halide exchange reactions using zinc iodide. PL spectra of the initial $CsPbBr_3$ NCs sample dispersed in toluene (black curve) and the corresponding samples upon the addition of zinc iodide (red curve) in methanol dispersion (a) and toluene dispersion (b), respectively. The inset in both panels shows the photographs of NCs dispersions recorded under UV-lamp irradiation before and after halide exchange reactions.

## Ageing tests

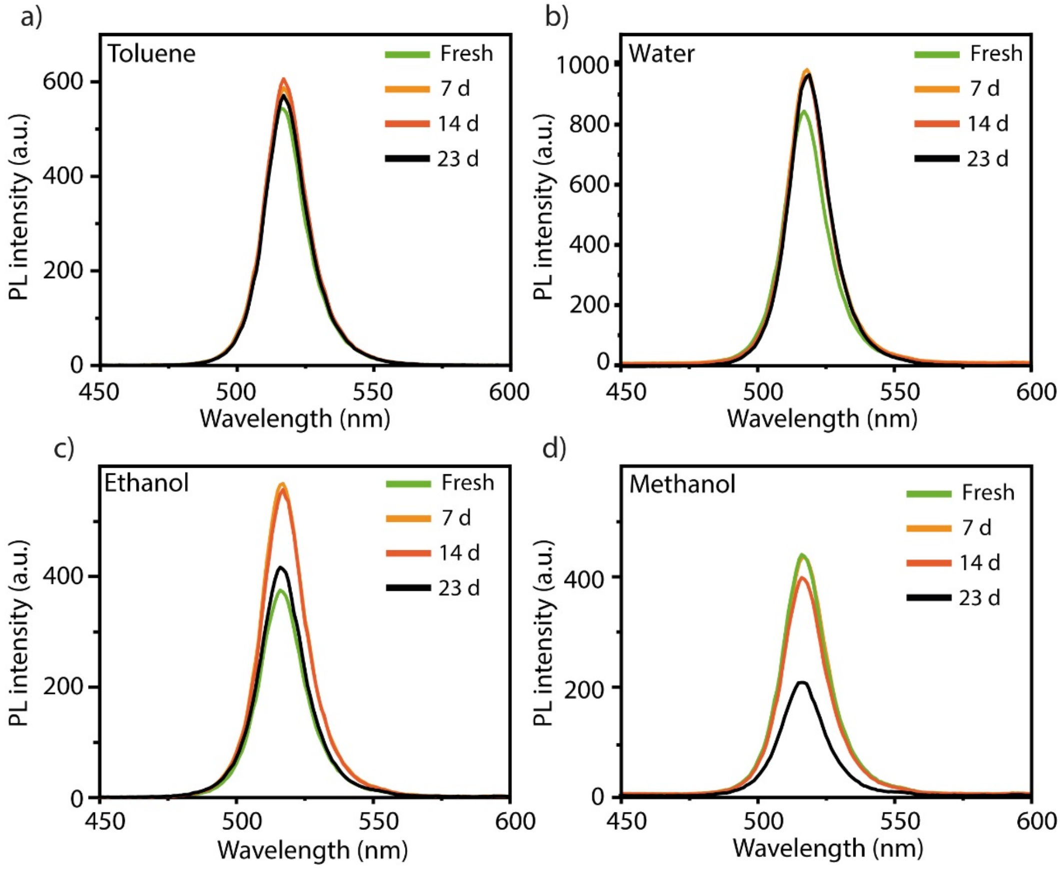


**Figure S10: Stability of PAA-b-PS encapsulated $CsPbBr_3$ NCs over ageing in different solvents.** (a) toluene, (b) water, (c) ethanol, (d) methanol. Samples were stored in cuvettes under ambient air and PL spectra were recorded over the period of 23 days.

## Detailed NMR analysis

We performed detailed liquid state nuclear magnetic resonance (NMR) spectroscopy to confirm that the micelles have an outer shell of PS and an inner core of PAA. The NMR measurements include $^{1}H$, $^{13}C$, 2D $^{1}H$-$^{13}C$, Heteronuclear Single Quantum Coherence (HSQC) and $^{1}H$-$^{13}C$ Heteronuclear Multiple Bond Correlation (HMBC). The experiments were performed on the starting free PAA-*b*-PS, empty PAA-*b*-PS micelles and PAA-*b*-PS encapsulated NCs. The free polymer sample was prepared by dissolving 8.25 mg of PAA-*b*-PS powder in 1mL of DMF-d7. The PAA-*b*-PS micelles sample was prepared by dissolving the same amount of polymer into DMF followed by drop-wise addition of the polymer solution into toluene to induce the micelle formation. Thereafter, the micelles were collected by adding hexane in excess to the crude solution, followed by centrifugation at 5000 rpm for 5 minutes. The supernatant was discarded and the dried precipitate was redispersed in d-8 toluene. The PAA-*b*-PS encapsulated NCs sample was prepared under the same conditions as those used for the empty micelles, but with the addition of metal

bromides ($CsBr$ and $PbBr_2$) and the solution containing the additive molecules. For NMR measurements, the samples were transferred into 5 mm disposable NMR tubes (Bruker).

We initially performed the $^1H$, $^{13}C$, 2D $^1H$-$^{13}C$, HSQC and $^1H$-$^{13}C$ HMBC NMR analysis on the starting free PAA-*b*-PS in DMF-$d_7$ as a solvent, see the results in Figure 3 c, d and Figure S11. DMF is a non-selective solvent for the polymer, therefore both the PAA and PS components of the polymer should be detectable through $^1H$ and $^{13}C$ NMR. The $^1H$ NMR analysis clearly evidenced the resonances corresponding to OH (1) of the carboxylic acid group (δ 12.66 ppm) and the CH (3) in position α to the CO group (δ 2.49 ppm) of the PAA component, along with the aromatic region (δ from 6.38 to 7.69 ppm) of the PS part of the polymer. Analogously, the CO (2) group (δ 176.4 ppm) of the PAA block, as well as the aromatic (5-8) signals (δ from 147.4 to 125.3 ppm) of the PS moiety are detectable in the $^{13}C$ spectrum, see Figure 3d of the main text. Interestingly, the 2D $^1H$- $^{13}C$ HSQC spectra, along with the $^1H$-$^{13}C$ HMBC cross correlations, which unambiguously confirm the cross correlations between the peaks (3) and (4) and the CO group (2) of the acidic function of the PAA moiety, enabled us to provide a complete assignment of the $^1H$ and $^{13}C$ peaks. Then, we ran the same analysis on PAA-*b*-PS micelles made from same polymer (the preparation of both samples is described in the previous paragraph) in toluene d-8. The results of $^1H$, $^{13}C$, 2D $^1H$-$^{13}C$, HSQC and $^1H$-$^{13}C$ HMBC NMR are reported in Figure 3 c, d top panels and Figure S12. The characteristic signals the carboxylic proton COOH (1, δ from 13.0 to 11.0 ppm) and of the CH proton (3) in position α to the CO group (δ ~ 2.5 ppm), which are distinguishable footprints of the PAA block, are not detectable. This is in line with the $^{13}C$ spectrum, in which the signal of C=O (δ from 180.0 to 170.0 ppm) is not noticeable (Figure 3d, top panel). These evidences indicate that the PAA block has a very poor mobility in toluene (it remains inaccessible to the solvent) and the only contribution to the high resolution NMR peaks is due to the PS moiety. We further recorded the $^1H$ NMR spectrum of the PAA-*b*-PS encapsulated NCs in toluene-$d_8$ and compared it with the $^1H$ NMR spectrum of the empty micelles (see Figure S13). The spectrum of the empty PAA-b-PS micelles has peak profiles and signal widths at half height that are identical to those of the micelles with the NCs inside, thereby confirming the similar structure of the polymer micelles in both cases. These measurements overall confirm that PAA forms the core of the micelles encapsulating the perovskite NCs while PS forms the outer shell. Overall, our NMR analysis verifies the structural conformation of the polymer, which consists of PAA as a rigid core and a PS outer shell, the latter exposed to the solvent thus more flexible.

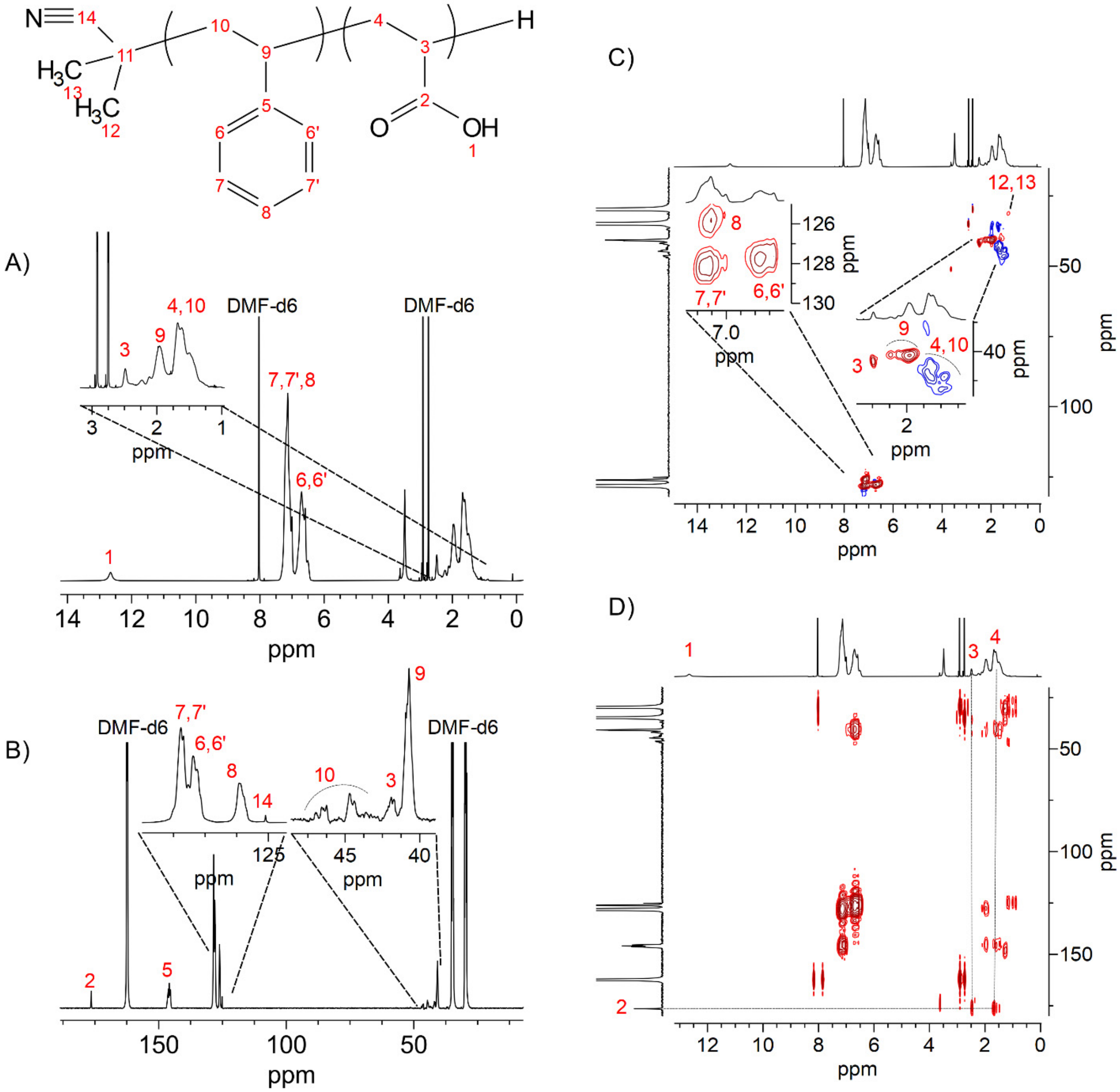


**Figure S11. $^1$H (A),$^{13}$C (B), $^1$H-$^{13}$C HSQC (C) and $^1$H-$^{13}$C HMBC (D) NMR spectra of the free PAA-b-PS polymer dissolved in DMF-d$_7$** The spectra reveal all the characteristic $^1$H and $^{13}$C peaks of the PAA-b-PS structure, along with their peak assignments, and $^1$H-$^{13}$C HMBC cross correlations. HMBC allows the possibility to identify the correlation between $^1$H and distant $^{13}$C (1-2 bonds). Here, HMBC confirm unambiguously the assignment of peak 3 of PAA moiety, through the HMBC cross correlations (dashed lines in the figure S11 D) between the signals 3 and 4, and the signal 2, whose $^{13}$C resonance (at 176.4 ppm) is uniquely attributed to a C=O group of the acidic function of PAA moiety.

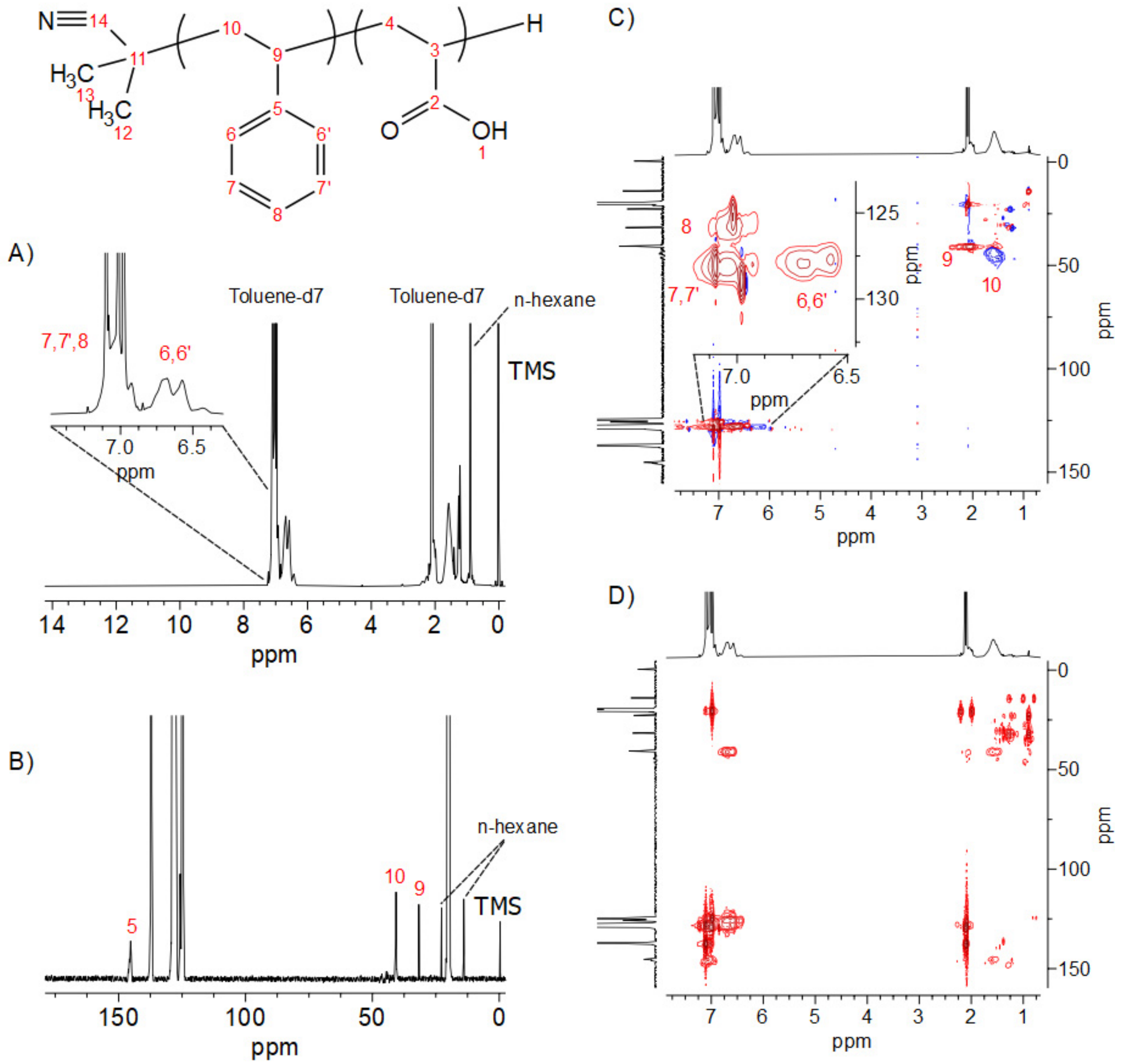


**Figure S12: $^{1}H$ (A),$^{13}C$ (B), $^{1}H$-$^{13}C$ HSQC (C) and $^{1}H$-$^{13}C$ HMBC (D) NMR spectra of empty PAA-b-PS micelles dispersed in toluene-$d_8$.** The spectra reveal all the characteristic $^{1}H$ and $^{13}C$ peaks of the PS structure along with their unambiguous peak assignments and $^{1}H$-$^{13}C$ correlation (2D NMR). In toluene-$d_8$, the $^{1}H$ and $^{13}C$ signals of PAA block are missing. These evidences indicate that PAA moiety has a poor mobility in toluene, which is consistent with a rigid core conformation.

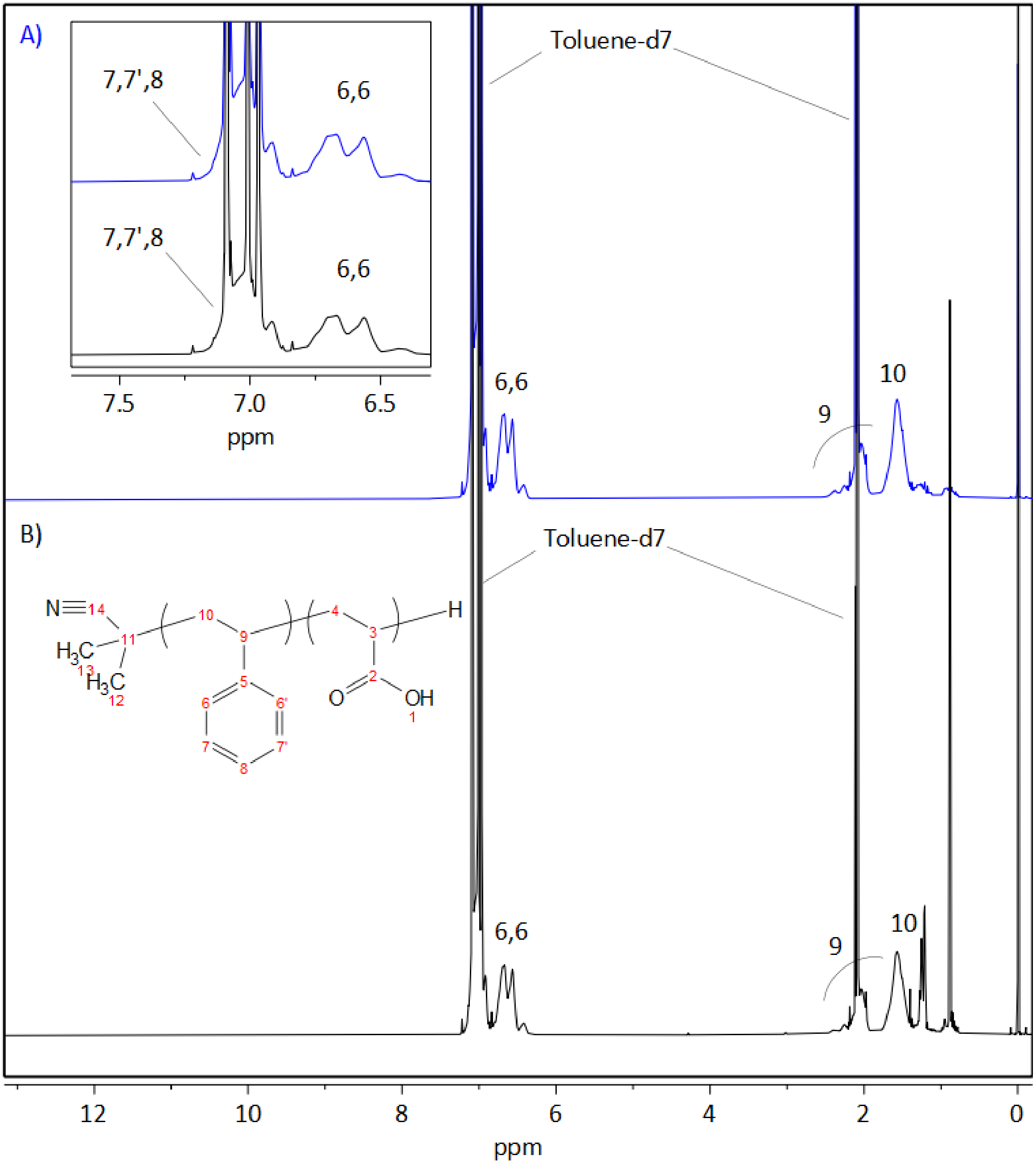


**Figure S13: the $^{1}$H NMR spectra of A) the PAA-*b*-PS encapsulated $CsPBBr_3$ NCs micelles and B) empty PAA-*b*-PS, in toluene-$d_8$.** The inset shows the aromatic region expanded. Broad aromatic peak corresponding to signals 7,7' and 8 of PS moiety of polymer, is clear distinguishable from not deuterated residual Tolene-d8 peaks (sharp).

## Stability tests using various additive molecules

To understand the role of the additive molecules, we initially prepared PAA-*b*-PS encapsulated $CsPbBr_3$, $Cs_{0.5}FA_{0.5}PbBr_3$ and $FAPbBr_3$ NCs by using two different organic molecules as additives, namely phenethylammonium bromide (PEABr, one functional group: ammonium) and 5-aminopentanoic acid (APAc, two functional groups: one carboxylic, one amino) and tested their stability against methanol. Freshly prepared samples were dispersed in methanol (under condition similar to those reported for Figure 3 of the manuscript) and the results are reported in Figure S10. The $CsPbBr_3$ and $Cs_{0.5}FA_{0.5}PbBr_3$ samples prepared using PEABr exhibit significant drop in the PL soon after their dispersion in methanol. The $FAPbBr_3$ sample suffered the most (nearly the complete loss of PL). On the other hand the samples prepared using APAc remained bright and stable in methanol. As the $FAPbBr_3$ NCs appeared to be the most sensitive to the type of molecule, we decided that an extensive search for a list of additive molecules that were good stabilizers for the polymer encapsulated NCs should be done indeed on NCs with $FAPbBr_3$ composition. The results of this search are reported in Figures S15-18).

PAA-*b*-PS encapsulated $FAPbBr_3$ NCs were prepared in the presence of various additive molecules having one, two or more functional groups, and either one aliphatic or one aromatic hydrocarbon chain, while all other reaction conditions were kept the same. Qualitative observations regarding the optical properties and stabilities of resulting PAA-*b*-PS encapsulated $FAPbBr_3$ NCs, when different additive molecules were used, are summarized in Table S4. Typical molecules that were tested include hexan-1-amine, hexanoic acid, 2-aminoethanethiol, 4-aminobutanoic acid, 5-aminopentanoic acid, (3-aminopropyl)phosphonic acid, 1,4-butandioic acid, 2-amino-3-hydroxypropanoic acid, pyrrolidine-2-carboxylic acid, 2-amino-3-methylbutanoic acid, 2-amino-5-(diaminomethylideneamino)pentanoic, 2-aminopentanedioic acid, -2-amino-3-sulfhydrylpropanoic acid, 2-amino-3-(1H-imidazol-4-yl)propanoic acid and 2-aminotertphthalic acid. Absorption and PL spectra measured in toluene dispersions are reported in Figure S15 and the photographs of the corresponding samples recorded under UV-lamp are reported in Figure S16. In toluene dispersions, the sample prepared using molecules having one amino and one thiol group (cysteine) had well defined absorption features but no PL at room temperature. Samples prepared using molecules containing one carboxylic, one thiol and one amino group or one carboxylic and two amino groups were weakly emissive (see Figure S15). Molecules containing more than one amino groups were not useful in the present case ($FAPbBr_3$ NCs), most likely due to their tendency to deprotonate the $FA^+$ cations, compromising the photoluminescence characteristic of corresponding samples, in agreement with previous reports.[8-9] Upon dispersing in methanol, the control sample (prepared without additive molecules) as well as the samples prepared using additive molecules with one functional group (amine, carboxylic acid and their mixture) significantly lost their PL. Notably, the sample prepared using a mixture of two additive molecules having each a single functional group attached to the hydrocarbon chain (hexan-1-amine + hexanoic acid) nearly lost its PL upon dispersion in methanol. On the other hand, the sample prepared using 5-aminopentanoic acid, which contains two functional groups (carboxylic and amino), both attached to the same hydrocarbon chain remains bright and stable. A comparison of these two latter samples shows that additive molecules containing two functional groups are much more effective than mixtures of two types of molecules each containing a single functional group. In general, the samples prepared by using molecules containing at least two or more functional groups remained bright and stable, see Figures S17-S18. The successful molecules had either all acidic (carboxylic, phosphonic) groups, or a

combination of acidic and at most one basic (such as amino) groups. Details on PL stability of PAA-*b*-PS encapsulated $FAPbBr_3$ NCs prepared by various additives molecules are summarized in Table S4.

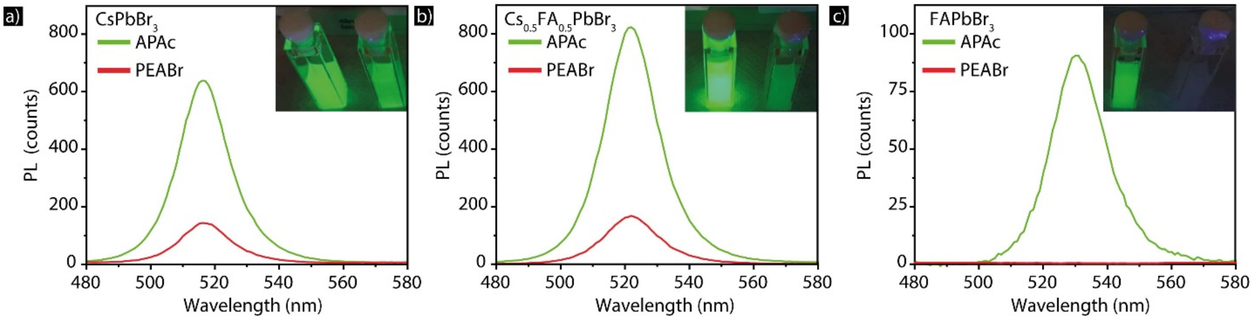


**Figure S14. Stability of PAA-b-PS encapsulated NCs.** (a) $CsPbBr_3$, (b) $Cs_{0.5}FA_{0.5}PbBr_3$ and (c) $FAPbBr_3$ NC samples prepared by using phenethylammonium bromide (PEABr) and 5-aminopentanoic acid (APAc) as additive molecules in methanol dispersions. The insets in panels are the photographs of the corresponding NCs samples under UV illumination (excitation, 365 nm).

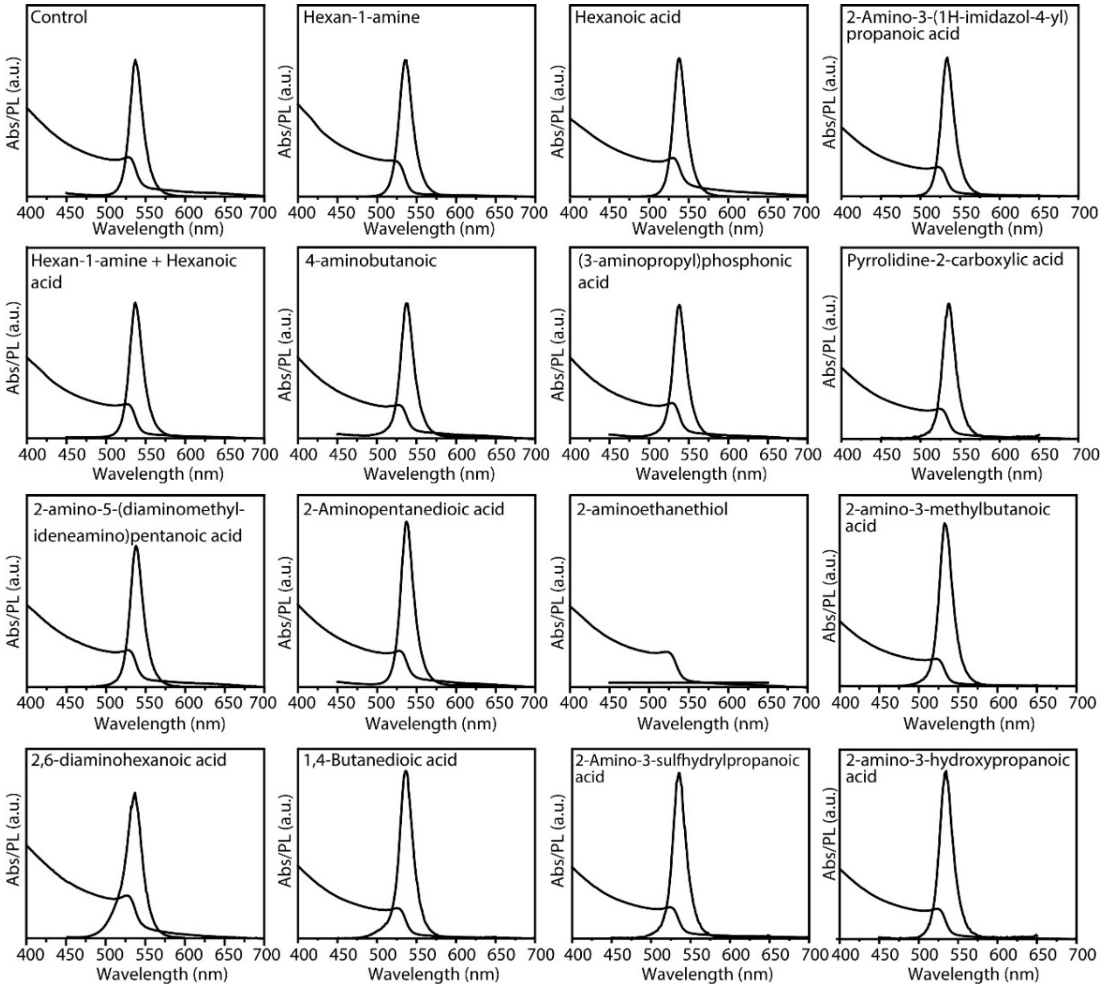


**Figure S15. Synthesis of PAA-*b*-PS encapsulated $FAPbBr_3$ NCs in the presence of additive molecules with different functional groups.** The control sample was prepared in the absence of additives. Optical absorption and PL spectra recorded were recorded in toluene dispersions for all the samples.

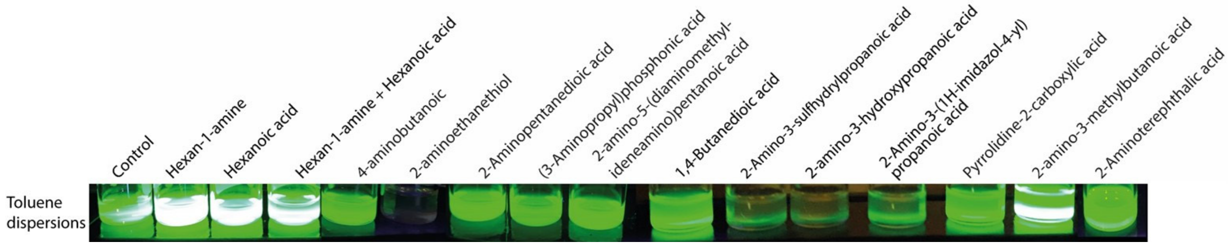


**Figure S16. Photograph of PAA-*b*-PS encapsulated $FAPbBr_3$ NCs dispersions in toluene under UV illumination (excitation, 365 nm).** the PL spectra of corresponding samples are reported in Figure S15.

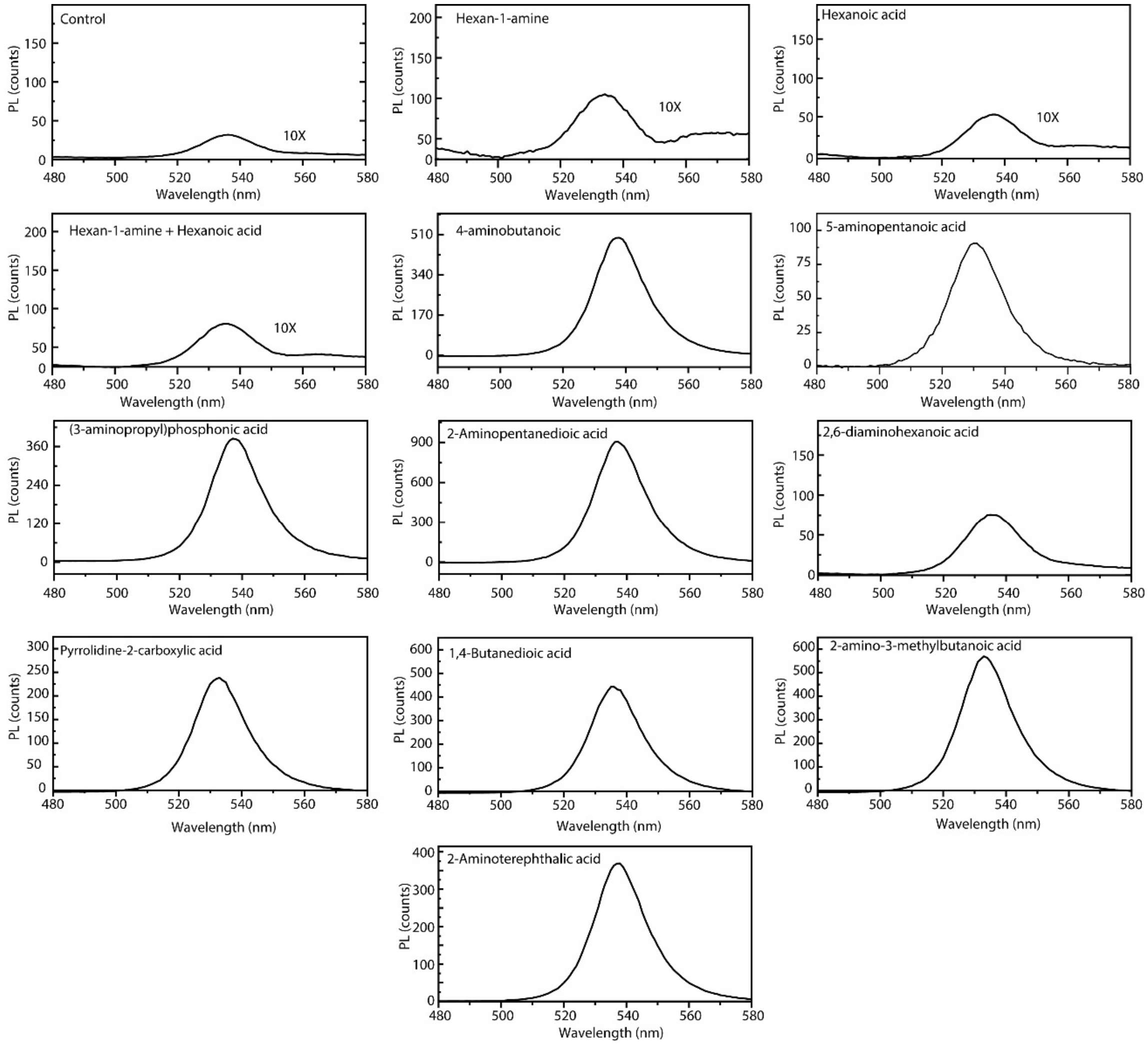


**Figure S17. Stability of PAA-b-PS encapsulated FAPbBr3 NCs prepared by using additive molecules with different functional groups in methanol.** PL spectra recorded on PAA-*b*-PS encapsulated $FAPbBr_3$ NCs prepared using various additive molecules (reported in Figure S15, S16) and dispersed in methanol.

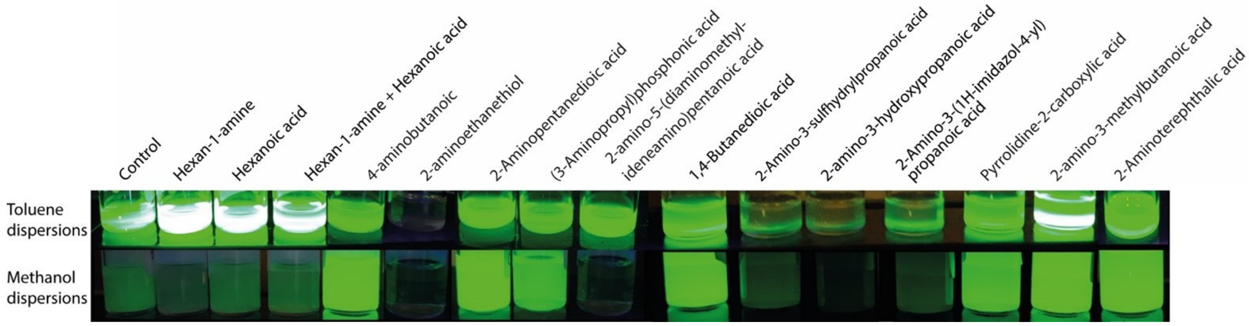


**Figure S18**. **The photograph of PAA-*b*-PS encapsulated $FAPbBr_3$ NCs samples in toluene and methanol dispersions under UV illumination (excitation, 365 nm).** The PL spectra of the samples in the corresponding solvents are reported in Figure S15 and S17.

**Table S4.** Optical properties of the PAA-*b*-PS encapsulated $FAPbBr_3$ NCs in terms of PL intensity and stability in toluene and methanol using different additives

| Additives | Number and nature of functional group | PL in toluene dispersions [a] | PL in Methanol dispersions [b] |
|---|---|---|---|
| No additives | - | Fair | Poor |
| hexan-1-amine | 1 (1 –$NH_2$) | High | Poor |
| Hexanoic acid | 1 (1 –COOH) | High | Poor |
| hexan-1-amine + Hexanoic acid | 1 (1 –$NH_2$) + 1 (1 –COOH) | High | Poor |
| 2-aminoethanethiol | 2 (1 –SH, 1 –$NH_2$) | No PL | - |
| 4-aminobutanoic | 2 (1 –COOH, 1 –$NH_2$) | High | High |
| 5-aminopentanoic acid | 2 (1 –COOH, 1 –$NH_2$) | High | High |
| (3-aminopropyl)phosphonic acid | 2 (1 –$P(=O)(OH)_2$, 1 $NH_2$) | High | High |
| 1,4-Butanedioic acid | 2 (2 –COOH) | High | High |
| 2-amino-3-hydroxypropanoic acid | 2 (1 –COOH, 1 -$NH_2$) | Poor | No PL |
| Pyrrolidine-2-carboxylic acid | 2 (1 –COOH, 1 –NH-) | High | High |
| 2-amino-3-methylbutanoic acid | 2 (1 –COOH, 1 -$NH_2$) | High | High |
| 2-amino-5-(diaminomethylideneamino)pentanoic acid | 2 (1 –COOH, 1 Formamidine) | High | No PL |
| 2-Aminopentanedioic acid | 3 (2 –COOH, 1 -$NH_2$) | High | High |
| 2-Amino-3-sulfhydrylpropanoic acid | 3 (1 –COOH, 1 -$NH_2$, 1 –SH) | Poor | No PL |
| 2-Amino-3-(1H-imidazol-4-yl)propanoic acid | 3 (1 –COOH, 1 –$NH_2$, 1 Imidazole) | Poor | Poor |
| 2-aminotertphthalic acid | 3 (2 –COOH, 1 –$NH_2$) | High | High |

[a,b] The sample were marked as Fair, poor and high based on their PL intensity in toluene and methanol dispersions. We used PAA-*b*-PS encapsulated $FAPbBr_3$ (5-aminopentanoic acid as additive) as a reference sample for the comparison of relative PL intensity. This sample has a PLQY of 61% and marked as "high" in terms of PL intensity. The samples prepared by using various additive molecules with the PL intensity at least 80% or above compared to the reference sample were considered as "high". On the other hand, the samples with PL intensity below 30% compared to the reference sample were classified as "poor". The samples having the PL intensity in the range of 30-80 % were considered as "fair". For the stability in methanol, the samples retaining 50 % of their PL compared to their starting PL in toluene dispersion were considered as "high". The samples losing more than 90% of their PL intensity upon dispersion in methanol were considered as "poor" and the samples with PL intensity within these extremes were considered as "fair".

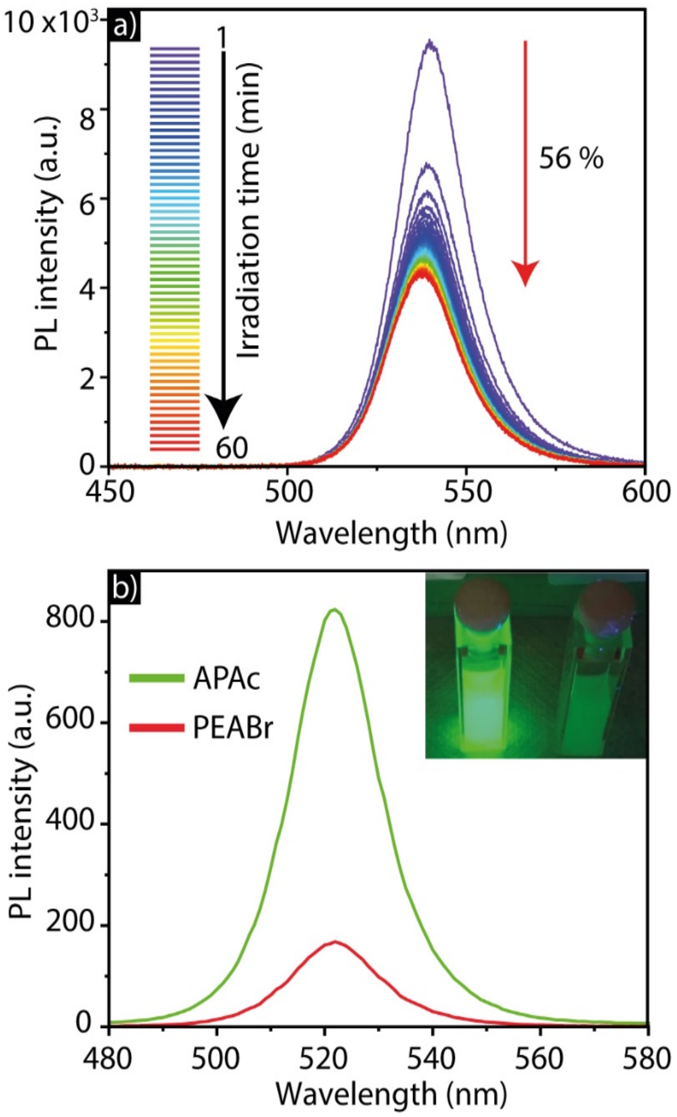


**Figure S19:** Stability of $Cs_{0.5}FA_{0.5}PbBr_3$ NCs prepared using two different additive molecules. (a) Evolution of PL spectra of NCs prepared by using phenethylammonium bromide (PEABr) showing 56 % loss of PL over 60 min of continuous exposure to laser irradiation. While instead, the samples prepared by using 5-aminopentanoic acid (APAc) evidenced 6% loss of PL for the same period of exposure to laser irradiation (see Figure 4d of the main text). Panel (b) shows the stability of both samples in methanol dispersions. The inset in panel (b) corresponds to the photograph of the same samples under UV illumination (excitation, 365 nm).

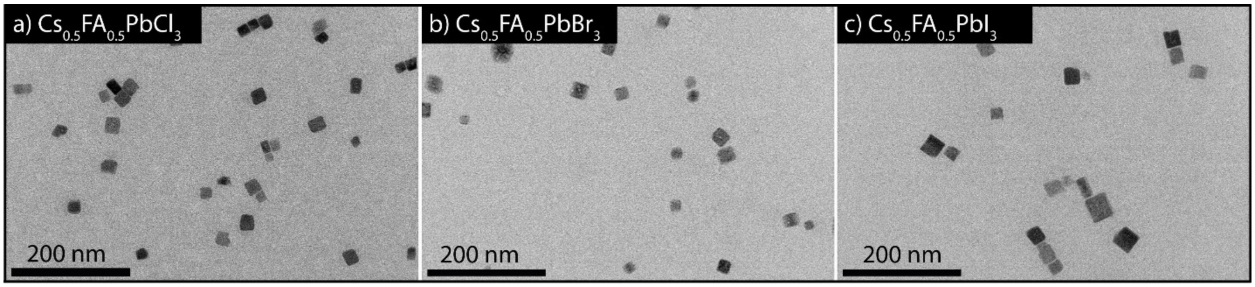


**Figure S20: Representative TEM images of PAA-*b*-PS encapsulated NCs.** (a) $Cs_{0.5}FA_{0.5}PbCl_3$, (b) $Cs_{0.5}FA_{0.5}PbBr_3$ and (c) $Cs_{0.5}FA_{0.5}PbI_3$ NCs deposited from toluene dispersions. $Cs_{0.5}FA_{0.5}PbCl_3$ and $Cs_{0.5}FA_{0.5}PbI_3$ NCs were prepared by halide exchange reactions starting from $Cs_{0.5}FA_{0.5}PbBr_3$ NCs.

## Additional data on emitting powders and on the white light emitting layer preparation

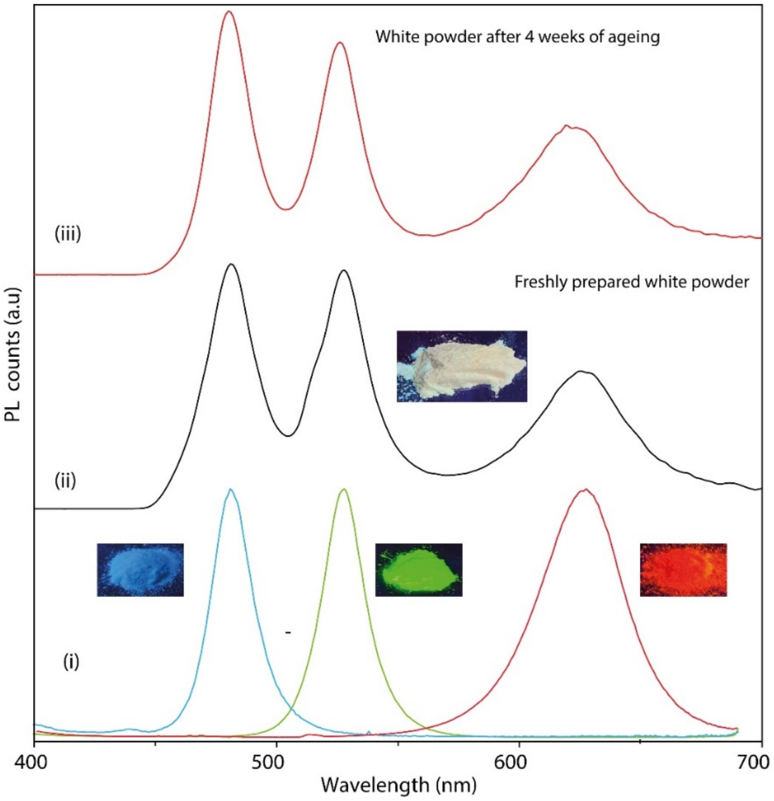


**Figure S21:** Comparison of the spectral features of PAA-*b*-PS encapsulated $Cs_{0.5}FA_{0.5}PbX_3$ NCs powders before (i) and after mixing (ii), evidencing the retention of their native PL peak position in the white emitting blend. A PL spectrum recorded the same powder mixture after 4 weeks of ageing under ambient air shows that the PL peak positions and relative PL intensities remain unchanged (iii). The insets correspond to the photographs of the various samples under UV illumination (excitation light at 365 nm).

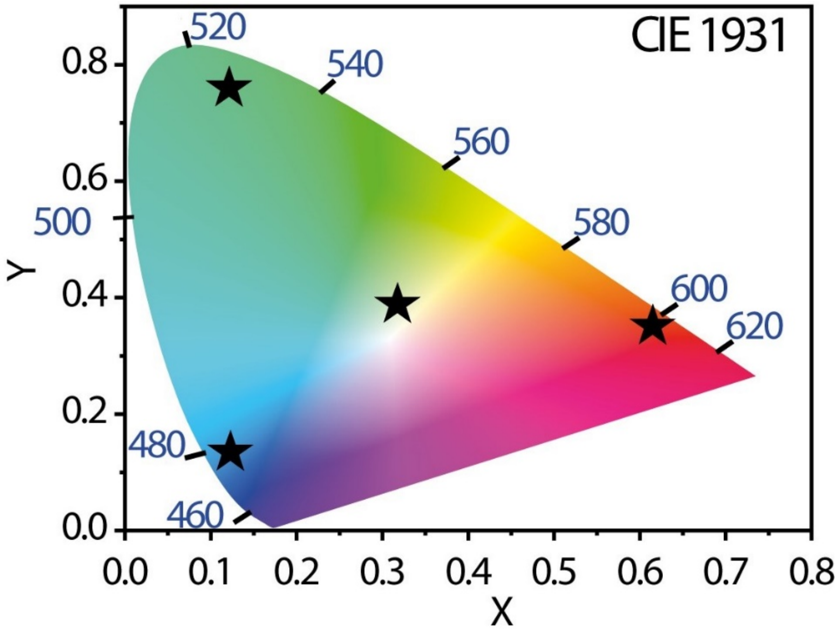


**Figure S22:** CIE 1931 diagram of the white emitting mixture of powders and the respective components reported in Figure 6 and Figure S16.

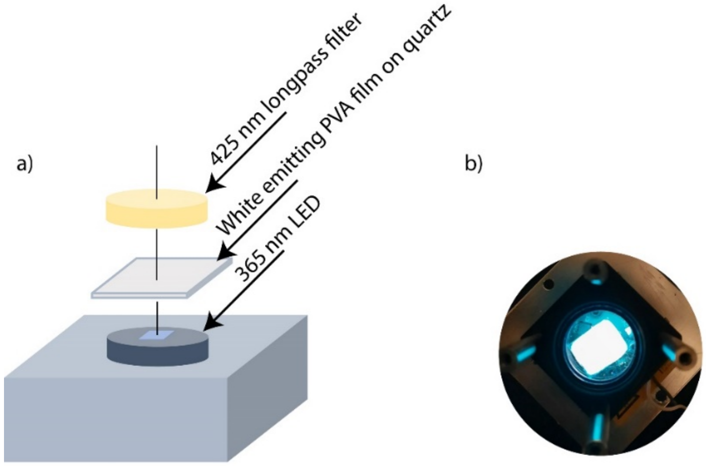


**Figure S23:** a) Schematic illustration of the device structure used for the fabrication of a white light-emitting device based on a UV-LED for excitation, a PVA film embedding the NCs and long pass filter. Panel (b) is a photograph of the white emitting device under operational conditions.

## References


1. Akkerman, Q. A.; Martínez-Sarti, L.; Goldoni, L.; Imran, M.; Baranov, D.; Bolink, H. J.; Palazon, F.; Manna, L., Molecular Iodine for a General Synthesis of Binary and Ternary Inorganic and Hybrid Organic–Inorganic Iodide Nanocrystals. *Chem. Mater.* **2018,** *30* (19), 6915-6921.
2. Zhang, J.; Jiang, P.; Wang, Y.; Liu, X.; Ma, J.; Tu, G., In-situ Synthesis of Ultrastable $CsPbBr_3$ Perovskite Nanocrystals Coated with Polyimide in a CSTR System. *ACS Appl. Mater. Interfaces* **2019,** *12*, 3080-3085.
3. Protesescu, L.; Yakunin, S.; Bodnarchuk, M. I.; Krieg, F.; Caputo, R.; Hendon, C. H.; Yang, R. X.; Walsh, A.; Kovalenko, M. V., Nanocrystals of cesium lead halide perovskites ($CsPbX_3$, X= Cl, Br, and I): novel optoelectronic materials showing bright emission with wide color gamut. *Nano Lett.* **2015,** *15* (6), 3692-3696.
4. Hou, S.; Guo, Y.; Tang, Y.; Quan, Q., Synthesis and stabilization of colloidal perovskite nanocrystals by multidentate polymer micelles. *ACS Appl. Mater. Interfaces* **2017,** *9* (22), 18417-18422.
5. Hintermayr, V. A.; Lampe, C.; Löw, M.; Roemer, J.; Vanderlinden, W.; Gramlich, M.; Böhm, A. X.; Sattler, C.; Nickel, B.; Lohmüller, T., Polymer nanoreactors shield perovskite nanocrystals from degradation. *Nano Lett.* **2019,** *19* (8), 4928-4933.
6. He, Y.; Yoon, Y. J.; Harn, Y. W.; Biesold-McGee, G. V.; Liang, S.; Lin, C. H.; Tsukruk, V. V.; Thadhani, N.; Kang, Z.; Lin, Z., Unconventional route to dual-shelled organolead halide perovskite nanocrystals with controlled dimensions, surface chemistry, and stabilities. *Sci. Adv.* **2019,** *5* (11), eaax4424.
7. Liu, Y.; Wang, Z.; Liang, S.; Li, Z.; Zhang, M.; Li, H.; Lin, Z., Polar Organic Solvent-Tolerant Perovskite Nanocrystals Permanently Ligated with Polymer Hairs via Star-like Molecular Bottlebrush Trilobe Nanoreactors. *Nano Lett.* **2019,** *19* (12), 9019-9028.
8. Protesescu, L.; Yakunin, S.; Bodnarchuk, M. I.; Bertolotti, F.; Masciocchi, N.; Guagliardi, A.; Kovalenko, M. V., Monodisperse formamidinium lead bromide nanocrystals with bright and stable green photoluminescence. *J. Am. Chem. Soc.* **2016,** *138* (43), 14202-14205.
9. Imran, M.; Caligiuri, V.; Wang, M.; Goldoni, L.; Prato, M.; Krahne, R.; De Trizio, L.; Manna, L., Benzoyl halides as alternative precursors for the colloidal synthesis of lead-based halide perovskite nanocrystals. *J. Am. Chem. Soc.* **2018,** *140* (7), 2656-2664.
